# Data-driven capacity estimation of commercial lithium-ion batteries from voltage relaxation

**DOI:** 10.1038/s41467-022-29837-w

**Published:** 2022-04-27

**Authors:** Jiangong Zhu, Yixiu Wang, Yuan Huang, R. Bhushan Gopaluni, Yankai Cao, Michael Heere, Martin J. Mühlbauer, Liuda Mereacre, Haifeng Dai, Xinhua Liu, Anatoliy Senyshyn, Xuezhe Wei, Michael Knapp, Helmut Ehrenberg

**Affiliations:** 1 Clean Energy Automotive Engineering Center, School of Automotive Engineering, Tongji University, 201804 Shanghai, China; 2 Institute for Applied Materials (IAM), Karlsruhe Institute of Technology (KIT), 76344 Eggenstein-Leopoldshafen, Germany; 3 Department of Chemical and Biological Engineering, University of British Columbia, Vancouver, BC V6T 1Z3 Canada; 4 Technische Universität Braunschweig, Institute of Internal Combustion Engines, Hermann-Blenk-Straße 42, 38108 Braunschweig, Germany; 5 School of Transportation Science and Engineering, Beihang University, 100083 Beijing, China; 6 Heinz Maier-Leibnitz Zentrum (MLZ), Technische Universität München, Lichtenbergstr. 1, 85748 Garching b, München, Germany

**Keywords:** Batteries, Electrical and electronic engineering, Materials for energy and catalysis, Scientific data

## Abstract

Accurate capacity estimation is crucial for the reliable and safe operation of lithium-ion batteries. In particular, exploiting the relaxation voltage curve features could enable battery capacity estimation without additional cycling information. Here, we report the study of three datasets comprising 130 commercial lithium-ion cells cycled under various conditions to evaluate the capacity estimation approach. One dataset is collected for model building from batteries with LiNi_0.86_Co_0.11_Al_0.03_O_2_-based positive electrodes. The other two datasets, used for validation, are obtained from batteries with LiNi_0.83_Co_0.11_Mn_0.07_O_2_-based positive electrodes and batteries with the blend of Li(NiCoMn)O_2_ - Li(NiCoAl)O_2_ positive electrodes. Base models that use machine learning methods are employed to estimate the battery capacity using features derived from the relaxation voltage profiles. The best model achieves a root-mean-square error of 1.1% for the dataset used for the model building. A transfer learning model is then developed by adding a featured linear transformation to the base model. This extended model achieves a root-mean-square error of less than 1.7% on the datasets used for the model validation, indicating the successful applicability of the capacity estimation approach utilizing cell voltage relaxation.

## Abstract (web summary)

Accurate capacity estimation is crucial for lithium-ion batteries' reliable and safe operation. Here, the authors propose an approach exploiting features from the relaxation voltage curve for battery capacity estimation without requiring other previous cycling information.

## Introduction

Lithium-ion batteries have become the dominant energy storage device for portable electric devices, electric vehicles (EVs), and many other applications^1^. However, battery degradation is an important concern in the use of lithium-ion batteries as its performance decreases over time due to irreversible physical and chemical changes^2,3^. State of Health (SoH) has been used as an indicator of the state of the battery and is usually expressed by the ratio of the relative residual capacity with respect to the initial capacity^4^. The accurate battery capacity estimation is challenging but critical to the reliable usage of the lithium-ion battery, i.e., accurate capacity estimation allows an accurate driving range prediction and accurate calculation of the maximum energy storage capability in a vehicle. Typically, the battery capacity is gained by a full discharge process after it has been fully charged. In a real-life usage scenario, the battery full charge is often achieved while the EVs are parking with grid connection, however, the battery discharge depends on the user behavior with uncertainties in environmental and operational conditions, a complete discharge curve is seldom available for on-board battery health monitoring. The battery charging and discharging voltage, as one of the easily obtained parameters, depend on both, thermodynamic and kinetic characteristics of the battery. Thus, those methods using a charge/discharge process are proposed to estimate capacity for practical applications^5,6^, in which the input variables are extracted from the measured voltage curves, and the data-driven methods using statistical and machine learning techniques have been popular in battery research recently due to their strong data processing and nonlinear fitting capabilities^6^^,^^7^. The data-driven methods do not need a deep understanding of battery electrochemical principles, but large numbers of data are required to ensure the reliability of model^8^. Severson et al.^9^ reported a promising route using machine learning to construct models that accurately predicted graphite ||LiFePO_4_ (LFP) commercial cell lives using charge-discharge voltage data. Zhang et al.^10^ identified battery degradation patterns from impedance spectroscopy using Gaussian process machine learning models. Ding et al.^11^ introduced a machine learning method for the improvement of the efficiency of membrane electrode assembly design and experiment. Such data-driven methods focus on the relationships among the input and output features, and a key part of data-driven battery state estimation is the extraction of degradation features, which largely determines the estimation performance^12–14^.

In practical electric transport applications, battery charging is essential and happens regularly compared to the random discharge process affected by the driving behaviors and road environments. Therefore, extracting voltage features from the charging process has attracted wide attention. Taking into account the state-of-the-art literature, three classes of voltage-based extraction methods can be defined: (I) CC (constant current) charge voltage-based, (II) CC-CV (constant current–constant voltage) charge voltage-based, and (III) rest voltage-based as listed in Supplementary Table 1. The partial charge process in a specific voltage range for feature extraction is commonly used for capacity estimation^15^, and the estimation accuracy of the state of art is ranging from a root-mean-square error (RMSE) of 0.39% to a RMSE of 4.26% based on in-house experiments and different public datasets^5,6,16^. The transformations of the partial voltage curves, i.e., differential voltage analysis^17,18^ and incremental capacity analysis^19–21^, are used for battery aging mechanism identification and capacity fade evaluation. Typically, SVR (Support Vectors Regression)^22^, GPPF (Gaussian Process Particle Filter)^23^, BPNN (Back-Propagation Neural Network)^24^, and linear model^20^ are applied to estimate battery capacity using the partial incremental capacity curve. Compared to the charge voltage-based methods, studies extracting features from the rest voltage are few. A representative battery capacity estimation method utilizing the resting process was proposed by Baghdadi et al.^25^. They proposed a linear model to estimate battery capacity using the voltage after 30 min rest when the cell is fully charged, and the capacity estimation percentage error is ranging from 0.7 to 3.3% for three different commercial batteries. Schindler et al.^26^ and Lüders et al.^27^ took the voltage relaxation for the lithium plating detection in the battery capacity fade process. Qian et al.^28^ used an equivalent circuit model (ECM) to describe the voltage relaxation and found that the extracted parameters provided an evaluation of the battery SoH and aging mechanisms. Attidekou et al.^29^ modeled the battery capacity decay during rest periods at 100% SoC using a dynamic time constant derived from the resistor-capacitor (RC) network model. However, as the amount of RC links increases, the complexity of the ECM will increase accordingly, which makes it difficult to use in an on-board application^30^. Besides, the accuracy and robustness of capacity estimation are difficult to evaluate because of the differences in battery types and working conditions^8,9^.

It has been proven that the relaxation process including the relaxation voltage value at a specific time and the voltage curve during a specific period shows a relationship with the battery SoH^26–29,31^. From the review of battery charging studies^32–34^, the real-time data of EVs^35,36^, and a survey of real-world EV charging (Supplementary Note 1, Supplementary Table 2 and 3, and Supplementary Figs. 1 and 2), in addition to the CC charging strategy, the multistage current charging algorithm using a SoC dependent charging current is a promising method to maximize the charging efficiency. The start of charge for the EVs is normally distributed around intermediate SoCs as expected from the statistics^35,37,38^. The various multistage current charge strategies and the uncertain start of charge points bring difficulties to the acquirement of specific voltage ranges under constant current in the voltage-based methods. The relaxation after being fully charged is relatively unaffected by the charging process and is also easy to obtain since the battery is fully charged with high probability in real EV usage^35,37,38^, there is also no need for additional devices as the voltage data can be directly obtained from the battery management system. However, to the best of our knowledge, the relaxation voltage curve of the battery has not yet been studied systematically with machine learning methods for large-scale data from different battery types. Herein, an approach based on features extracted from the battery relaxation voltage is proposed, which focuses on short-term battery capacity estimation without any previous cycling information for on-board implementation.

In this study, base models using machine learning methods, i.e., the linear model (ElasticNet^39^), and nonlinear models (XGBoost^40^ and Support Vector Regression (SVR)^41^), using large datasets from three kinds of commercial lithium-ion batteries are employed. The model inputs are statistical features extracted from the voltage relaxation curve. Batteries with LiNi_0.86_Co_0.11_Al_0.03_O_2_ positive electrode (NCA battery) cycled at different temperatures and current rates are used for base model building, showing the best test performance with a RMSE of 1.0%. The transfer learning method is applied on batteries with LiNi_0.83_Co_0.11_Mn_0.07_O_2_ positive electrode (NCM battery) and batteries with 42 (3) wt.% Li(NiCoMn)O_2_ blended with 58 (3) wt.% Li(NiCoAl)O_2_ positive electrode (NCM + NCA battery), obtaining 1.7% RMSE and 1.6% RMSE respectively, and enabling the generalizability of our approach.

## Results

### Data generation

Large cycling datasets on NCA battery, NCM battery, and NCM + NCA battery are created in this study. The batteries are cycled in a temperature-controlled chamber with different charge current rates. The battery specifications are listed in Supplementary Table 4. Long-term cycling is conducted on all cells with a summary of cycling conditions in Table 1. The temperatures chosen are 25, 35, and 45 °C. Current rates ranging from 0.25 C (0.875 A) to 4 C (10 A) are used. The current rate is calculated from the nominal capacity of batteries, i.e., 1 C is equal to 3.5 A for the NCA battery and NCM battery, and 1 C is equal to 2.5 A for the NCM + NCA battery. The cells are named as CYX-Y/Z according to their cycling conditions. X means the temperature, Y/Z represents the charge/discharge current rate. The number of cells assigned to each cycling condition in Table 1 is aimed to obtain a dataset covering possible variations between cells. One data unit comprises a relaxation voltage curve after full charge with the following discharge capacity. Each relaxation voltage curve is transformed into six statistical features, i.e., variance (Var), skewness (Ske), maxima (Max), minima (Min), mean (Mean), and excess kurtosis (Kur). The mathematical description of the six features is depicted in Supplementary Table 5. The datasets collected from NCA, NCM, and NCM + NCA cells are named as dataset 1, dataset 2, and dataset 3 in this study, respectively. Dataset 1 is used for base model training and test. Dataset 2 and dataset 3 are used for assessing and improving the generalizability of the proposed approach by transfer learning.

**Table 1:** Cycled batteries and cycling conditions for the dataset generation.

Datasets	Cell type	Cycling temperature (±0.2 °C)	Charge current rate (C)/discharge rate (C)	Number of cells	Number of data units
Dataset 1	NCA battery	25	0.25/1	7	1853
	Type: 18,650		0.5/1	19	3278
	Cutoff voltage: 2.65–4.2 V		1/1	9	260
	Nominal capacity: 3.5 Ah	35	0.5/1	3	1112
		45		28	15,775
Dataset 2	NCM battery	25		23	5490
	Type: 18,650	35		4	4712
	Cutoff voltage: 2.5–4.2 V	45		28	17,600
	Nominal capacity: 3.5 Ah				
Dataset 3	NCM+NCA battery	25	0.5/1	3	2843
	Type: 18650		0.5/2	3	2913
	Cutoff voltage: 2.5–4.2 V		0.5/4	3	2826
	Nominal capacity: 2.5 Ah				

All cells are commercial 18,650 type batteries. The cycling temperature is controlled by climate chambers (±0.2 °C). The current rate is calculated from the battery nominal capacity (1C = 3.5 A for the NCA battery and NCM battery, and 1C = 2.5 A for the NCM + NCA battery).

**Fig. 1:**
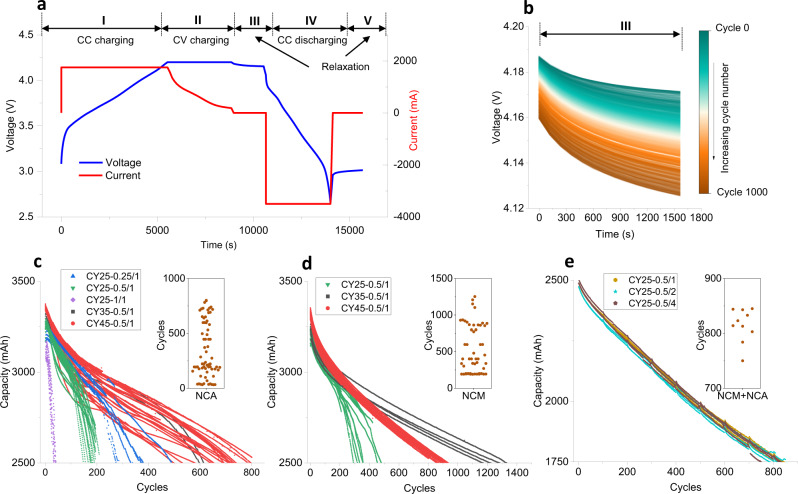
Battery cycling data. Voltage and current profile in the first cycle of one CY25-0.5/1 NCA battery (**a**). A plot of relaxation voltage change (region III) while cycling for one NCA cell (**b**). NCA battery discharge capacity (until 71% of nominal capacity) versus cycle number of NCA battery (**c**), NCM battery (**d**), and NCM+NCA battery (**e**). The embedded plots in **c**, **d**, and **e** are the cycle distribution of cells at around 71% of nominal capacity, the points are offset randomly in the horizontal direction to avoid overlapping.

Voltage and current are the basic data recorded in these experiments, which include charging, discharging, and relaxation processes. The cell cycling is performed with constant current (CC) charging to 4.2 V with current rates ranging from 0.25 C (0.875 A) to 1 C (3.5 A), followed by a constant voltage (CV) charging step at 4.2 V until a current of 0.05 C is reached. Constant current is then employed for the discharge to 2.65 V for the NCA cells and 2.5 V for the NCM and NCM + NCA cells, respectively. One complete cycling curve using a 0.5 C charging rate for the NCA cell is shown in Fig. 1a, which includes five processes, i.e., (I) CC charging, (II) CV charging, (III) relaxation after charging, (IV) CC discharging, and (V) relaxation after discharging. The CC discharging capacity is treated as the battery residual capacity during cycling. The relaxation time between the CV charging and CC discharging is 30 min for the NCA battery and NCM battery with a real sampling time of 120 s, and it is 60 min for the NCM + NCA battery with a sampling time of 30 s. The starting and ending voltage during the battery relaxation show a declining trend with increasing cycle number as presented in Fig. 1b.

Three datasets with capacity down to 71% of the nominal capacity are generated. The battery capacity as a function of cycle number for the NCA cells is shown in Fig. 1c. The cycle number is ranging from 50 to 800 in the 100–71% capacity window. It is evident that both, charging current and temperature have a strong influence on the capacity decay, and the battery capacity shows significant variance as depicted in the embedded plot in Fig. 1c, indicating the degradation distribution of the cycled cells. The worst scenario is the one with cells cycled at 1C charge at 25 °C (CY25-1/1), only 50 cycles can be obtained until the cells reach 71% of the nominal capacity. In all, 71% capacity is reached after 125 and 600 cycles at 25 and 35 °C respectively, for cells charged with 0.5 C (CY25-0.5/1, and CY35-0.5/1). In total, 71% capacity is reached after 250 cycles at 25 °C with 0.25 C charging current (CY25-0.25/1) and in a range of 500–800 cycles at 45 °C with 0.5 C charging current (CY45-0.5/1). The cycling data of the NCM cells are shown in Fig. 1d. Fatigue down to 71% residual capacity is found between 250 and 500 cycles (25 °C), 1250 and 1500 cycles (35 °C), and around 1000 cycles at 45 °C cycling temperature. The capacity fade results indicate that increasing the temperature to 35 and 45 °C has a beneficial effect on the capacity retention and that the charging current is at the limit of what the cells can handle. For NCA and NCM cells, a capacity spread for the cells cycled under equal conditions is observed, which is speculated to be ascribed to the intrinsic manufacturing variations as this spread is already seen at the beginning of cycling^42,43^. The cycling data of the NCM + NCA cells are shown in Fig. 1e, exhibiting a linear degradation trend regardless of the cycling discharge rates, and 71% residual capacity appears in a range of 750 to 850 cycles showing the influence of the cell cycling conditions.

### Feature extraction

**Fig. 2:**
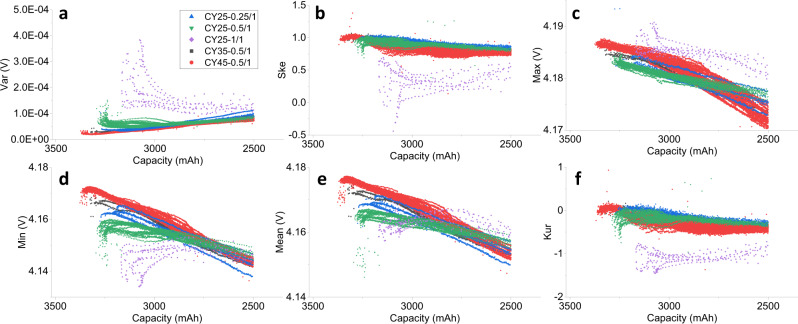
Extracted features from the voltage relaxation curves as a function of battery capacity for NCA cells. (**a**) Variance (Var), (**b**) skewness (Ske), (**c**) maxima (Max), (**d**) minima (Min), (**e**) mean (Mean), and (**f**) excess kurtosis (Kur). Feature changes between 3500 mAh and 2500 mAh (71% of nominal capacity) for NCA cells are shown to be consistent with the used datasets. The mathematical description of the six features is depicted in Supplementary Table 5.

Summarizing statistics are proven to be effective to illustrate numerically the shape and position change of the voltage curve^5,9^. As mentioned above, the relaxation process after fully charging is taken for feature extraction because of its strong relationship with battery degradation and its easy acquisition in battery real use. Each voltage relaxation curve is converted to six statistical features, i.e., Var, Ske, Max, Min, Mean, and Kur, as displayed in Fig. 2.

The relationship between battery capacity and the corresponding features is dependent on the cycling conditions as presented in Fig. 2. It can be seen that it is difficult to describe the relationships only by linear functions. The Var in Fig. 2a represents the distribution of the voltage points in one relaxation process, a decrease of Var versus capacity fade means that the relaxation voltages show a sharper distribution with increasing cycle number, and vice versa. Both Ske and Kur are normalized using Var, they are used to describe the shape of the corresponding voltage curve. The Ske in Fig. 2b is positive for almost all cycling conditions, indicating that more than half of the sampled voltage data are below the average voltage (Mean), which corresponds to the shape of the relaxation voltage curve, i.e., with respect to the relaxation time, the voltage drops initially fast and then gradually slows down. The Max in Fig. 2c presents a monotonous decrease of the maximum voltage versus capacity drop for all cycling conditions. The Min and Mean first increase and then decrease versus the capacity reduction as displayed in Fig. 2d, e, respectively. The Kur shown in Fig. 2f is the excess kurtosis obtained from the kurtosis of the raw data minus the kurtosis of a normal distribution. The excess kurtosis is negative for all cycling conditions, meaning that the distribution of the relaxation voltage is gentler than a normal distribution.

### Capacity estimation

**Fig. 3:**
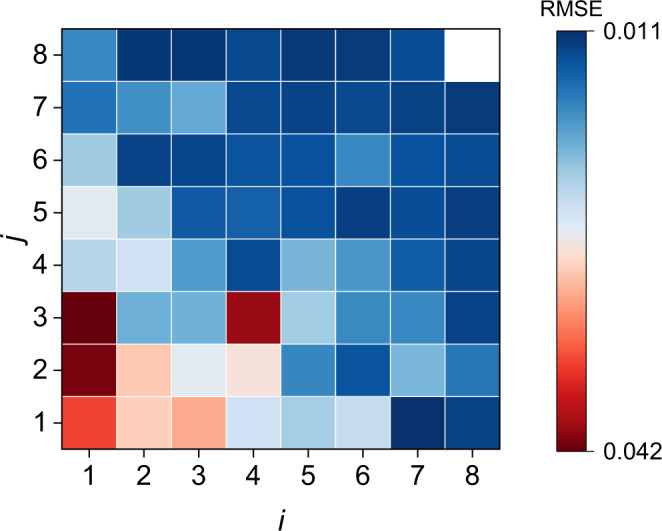
Cross-validation root-mean-square error (RMSE) of the XGBoost method using different feature combinations. (*i, j*) means different feature combinations referring the Supplementary Table 10. The (*7, 1*) = [Var, Ske, Max] obtains the best cross-validation RMSE = 1.0% within a three feature combination.

Based on the features extracted from the relaxation voltage curve after charging, data-driven methods are used for battery capacity estimation. Owing to the difference in the order of magnitudes of the features, a standard normalization for battery features is performed for dataset 1. The features of dataset 2 and dataset 3 are normalized by applying the same normalizing scales as used for dataset 1. The capacity is uniformized considering the difference in the battery nominal capacity. The XGBoost^40^ is selected as the main machine learning method. The ElasticNet^39^ as the multivariate linear model is used for comparison, and the SVR^41^ is a support for the verification of the transfer learning approach. For the base model training and test, different data splitting strategies are compared with dataset 1 in Supplementary Note 2 and Supplementary Tables 6–9. The best test result of the temperature dependence splitting method shows a 1.5% RMSE. A 2.3% test RMSE is obtained from the time-series data splitting method. The data random splitting and cell stratified sampling methods achieve good estimation accuracy with 1.1% RMSEs, implying that the variation of the working conditions leading to different degradation patterns is essential to improve the generalization of the model. The results of cell stratified sampling method meaning that the data from the same cell is either in the training set or in the test set are presented in this study (Strategy D in Supplementary Note 2). The cells are approximately in a 4:1 ratio for training and test (Supplementary Table 9). In the model training process, the K-fold cross-validation with *K* = 5 is used to determine the hyperparameters of the models. A feature reduction is performed by using different feature combinations to reduce the number of inputs and simplify the model complexity. The cross-validation RMSEs under different feature combinations using the XGBoost method are compared in Fig. 3. The *i* and *j* are used to represent different feature combinations referring the Supplementary Table 10.

**Fig. 4:**
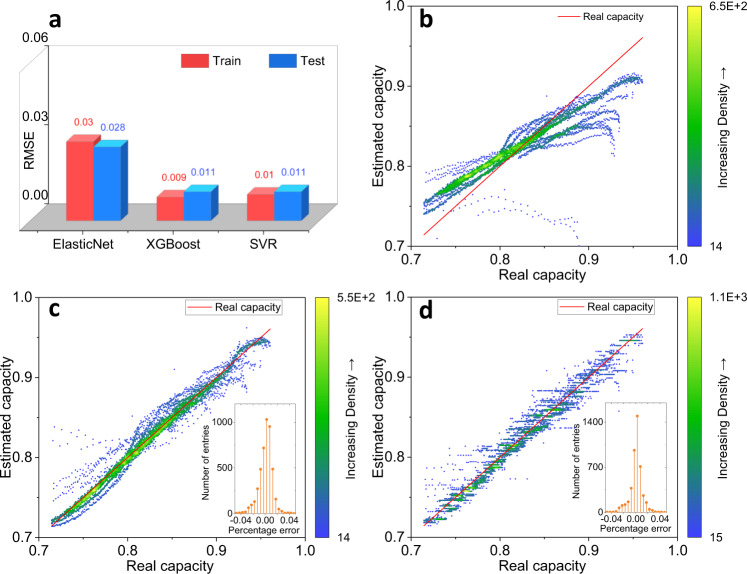
Results of battery capacity estimation with the input of three features [Var, Ske, Max] by different estimation methods. The capacity results are uniformized by the nominal capacity for comparison. root-mean-square error (RMSE) of battery capacity estimation (**a**), test results of estimated capacity versus real capacity by ElasticNet (**b**), XGBoost (**c**), and Support Vectors Regression (SVR) (**d**).

It shows that the RMSE gradually decreases as the number of features increases, and the accuracy improvement is no longer obvious after using three features in Fig. 3. The best estimation result is obtained by the input [Var, Ske, Max] in a three feature combination. The effect of the duration of the relaxation on the capacity estimation is presented in Supplementary Fig. 3, in which the RMSEs of training and test decrease as the relaxation time increases in the XGBoost method, indicating that longer relaxation time improves the model accuracy. Therefore, the Var, Ske, and Max of the voltage relaxation after 30 min are extracted as inputs for the base model. The hyperparameters of each algorithm are available in Supplementary Table 11. The RMSEs of different estimation methods on dataset 1 are summarized in Fig. 4a. It can be concluded that the test RMSE of XGBoost and SVR all reaches 1.1%, showing better performance than the linear model, and the RMSEs of train and test are close to each other, indicating the effectiveness of data splitting. The estimated capacity versus real capacity is illustrated in Fig. 4b–d for visualization purposes.

### Performance of the proposed approach

The performance of the proposed approach is benchmarked with state-of-the-art models using voltage curves for battery capacity estimation as shown in Table 2. One representative method is selected from each class of the presented capacity estimation methods (Supplementary Table 1). Since the datasets used in the literature are different in battery material and test procedures from ours, the strategy to solve this difference is to apply their algorithms to our datasets. A detailed description of data processing and estimation results for each method is presented in Supplementary Note 3 and Supplementary Figs. 4–7. The performance of the linear model to estimate the battery capacity based on the resting voltage in Baghdadi et al.^25^ shows a 2.5% RMSE, which can be explained by the large data volume and variety of working conditions in our dataset 1 highlighting the difficulty of capacity estimation only with the linear model. In the CC charge voltage-based methods, the random forest regression (RFR) method^16^ using the voltage ranging from 3.6 V to 3.8 V achieves a RMSE of 1.0% on dataset 1, which is 0.1% less than our RMSE based on the voltage relaxation. A method based on the remaining electrical charge with a threshold according to the incremental capacity value is proposed in Peri et al.^20^. The application of the same incremental capacity transformation method on dataset 1 provides a RMSE of 1.3%, indicating that our proposed approach has better accuracy. The Gaussian process regression (GPR) method^44^ using a full CC-CV charge voltage curve obtains good estimation results on dataset 1 with a test RMSE of 1.1%. Compared with the current research status, especially with respect to large datasets, the proposed approach using resting voltage can achieve a good estimation accuracy. As mentioned in the introduction section, there are some challenges in the acquisition of specific charging voltage curves because the start of battery charge is usually dependent on the driver behavior and the charge modes differ significantly from the charging stations in the real application of EVs. The relaxation process of a battery being fully charged is easily obtained without the requirement of specific working conditions and voltage ranges, which offers a new sight for battery capacity estimation.

**Table 2:** Test means root-mean-square error (RMSE) of different models using voltage-based features for battery capacity estimation.

Features from	Methods	Test RMSE on Dataset 1
Rest voltage-based	Linear model^25^	0.025
Constant current charge voltage-based	Random forest regression^16^	0.010
Incremental capacity analysis transformation	Linear model^20^	0.013
Constant current–constant voltage charge voltage-based	Gaussian process regression^44^	0.011

### Physical explanation

The alternating current (AC) electrochemical impedance provides information in the frequency domain on the degradation mechanisms of the battery as proven in ref. ^45^. The degradation mechanisms can be determined from the change of electrochemical impedance parameters extracted by fitting the impedance spectra with an ECM^46^. A schematic plot of electrochemical impedance spectra during cycling and the corresponding ECM are complemented in Supplementary Figure 8. Basically, an increase of R0 is likely due to contact loss and the reduction of ionic conductivity in the electrolyte^47^. R1 represents the resistance associated with the anode solid electrolyte interphase (SEI) indicated by the semicircle at high frequencies^46^. R2 is the charge-transfer resistance describing the rate of electrochemical reaction, which is related to the loss of electrode material through particle cracking^18,48^. The capacity loss of the cycled cells in dataset 1 and dataset 2 has been investigated by in situ neutron powder diffraction in our previous work^42^, which exhibits that the decrease in lithium content in the positive and negative electrodes correlates well with the observed discharge capacity. Both positive and negative electrodes do not decompose to other crystalline phases during cycling, but the lithium loss in the electrodes leading to lithiated material loss is traced by detecting changes in the lattices of the electrodes. The lithiated material loss and the SEI formation are suspected to contribute to the lithium loss.

**Fig. 5:**
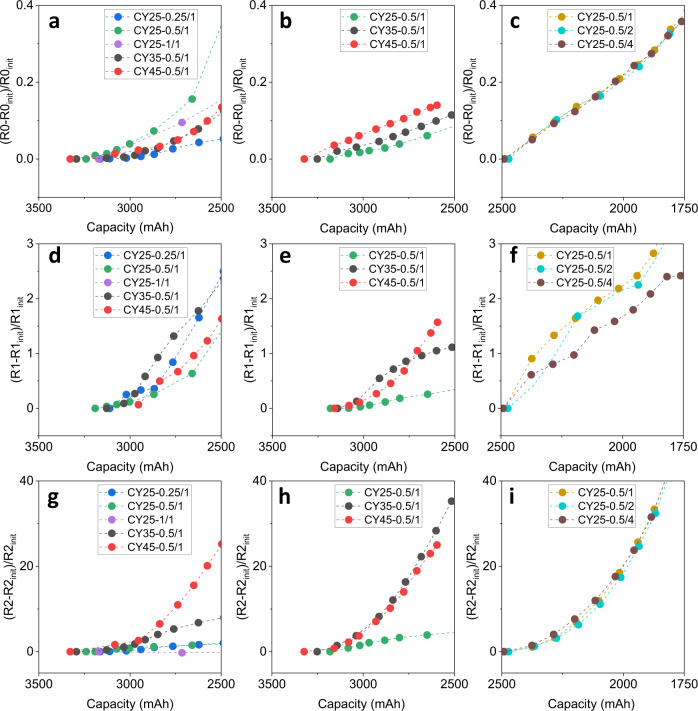
AC electrochemical impedance variations of the lithium-ion cells during cycling. The resistance increment from the initial value (*R*_init_) is calculated for comparison. The ohmic resistance of NCA cells (**a**), NCM cells (**b**), and NCA+NCM cells (**c**). SEI resistance of NCA cells (**d**), NCM cells (**e**), and NCA+NCM cells (**f**). Charge transfer resistance of NCA cells (**g**), NCM cells (**h**), and NCA+NCM cells (**i**). Only resistances before the capacity reducing to 71% of nominal capacity are shown to be consistent with the datasets in the study. The coefficient of determination (*R*^2^) between the raw and fitted impedance data is summarized in Supplementary Table 12. The SEI resistances are not identified in some cycles (seen in Supplementary Table 12) for the NCA battery (**d**) and NCM battery (**e**). The shared information of raw impedance data and fitted data can be found in the data availability.

Herein, the dominating aging factors for each cycling group are discussed by fitted electrochemical impedance parameters in Fig. 5. The coefficient of determination (R^2^) of each measured impedance spectrum between the raw and fitted data is summarized in Supplementary Table 12. All R^2^ values are greater than 0.999, indicating the credible fitting accuracy. All the raw and fitted impedance data can be found from the data availability. By comparison of the resistance increment from the initial value (R_init_) for all three type cells, the increment of R0 is minimal (Fig. 5a–c), followed by R1 (Fig. 5d–f). R2 shows the highest increase during the battery capacity fade as shown in Fig. 5g–i. The dominating degradation factors are different under different working conditions. For the NCA cell, as shown in Fig. 5a, the CY25-0.25/1 shows a steady and relatively small increase of R0, nevertheless, its R1 in Fig. 5d shows an accelerated rise, indicating the increase in the thickness of the SEI layer. The R2 of CY25-0.25/1 in Fig. 5g presents a similar increasing trend to its R0. The R0 of CY25-0.5/1 and CY25-1/1 in Fig. 5a remains the largest resistive contribution throughout, but their R1 and R2 are relatively lower than that of others, which indicates a more serious cell degradation such as electrolyte dry-out or contact loss likely caused by lithium plating^47,49^. For the results of NCM cells in Fig. 5b, e, h, all resistances of CY25-0.5/1 increase slowly, while resistances of cells cycled at 35 and 45 °C exhibit a large increase rate. For the NCA + NCM cells, the influence of discharge rate is mainly represented by R1 by comparing the results in Fig. 5c, f, i. The CY25-0.5/4 SEI resistance increase in Fig. 5f is significantly slower than that of other cycling conditions. The temperature influence on the degradation mechanism can be seen in Fig. 5g, h, in which the increase of R2 is associated mainly with the increase of ambient temperature. The cells cycled at 45 and 35 °C mainly lead to an increase of R2, which could be associated with the positive active material loss, e.g., particle cracking and pulverization^50,51^. The diversity of the battery internal degradation mechanisms results in various degradation paths, which can explain the difficulty in applying a simple linear model on the battery capacity estimation. Additionally, it seems that different battery types follow to some extent similar degradation rules, e.g., the exponential rise of R2, inspiring the use of transfer learning in the following part.

### Approach verification by transfer learning

The transfer learning (TL) method, which is applied to improve the learning ability by rebuilding the machine learning model using a relatively small amount of newly collected data, is proposed for easy adaption to the variation of voltage features existing in dataset 2 and dataset 3 in which different batteries and cycling conditions are used. The model weights are pre-trained through dataset 1 to obtain the base model. Then, some new data units from dataset 2 and dataset 3 are set as the input variable to re-train the TL model. Different data selection methods are discussed in Supplementary Note 4 and Supplementary Table 13, depicting that the variation of working conditions is necessary to improve the accuracy of the model estimation. One cell is randomly selected from each cycling condition in dataset 2 and dataset 3, then the data units in each cell are chosen with an interval of 100 cycles as the input variables for the re-training of TL models (Strategy D in Supplementary Note 4). The sizes of the input variable are summarized in Supplementary Table 14 (occupying 0.06% of dataset 2 and 0.35% of dataset 3). Verification on dataset 2 and dataset 3 without changing any weights of the base model is used as a zero-shot learning (ZSL) reference. The full base model is retrained using the same input variables from dataset 2 and dataset 3 as a No TL comparison. Two TL methods (TL1 and TL2) with fine-tuning strategies are activated to adjust the weights of a newly added layer, while the weights of other layers remain unchanged. TL1 means that a linear transformation layer is added before the output of capacity. TL2 means that a linear transformation layer before the base model is constructed to adapt the input features as illustrated in Supplementary Fig. 9. The test RMSEs are compared in Table 3.

**Table 3:** Test RMSEs of battery capacity estimation using zero-shot learning (ZSL) and different transfer learning (TL) methods on dataset 2 and dataset 3.

Methods	Dataset	ZSL	No TL	TL1	TL2
XGBoost	Dataset 2	0.038	0.029	0.027	0.024
	Dataset 3	0.038	0.020	0.034	0.024
Support vectors regression	Dataset 2	0.034	0.039	0.026	0.017
	Dataset 3	0.073	0.052	0.035	0.016

**Fig. 6:**
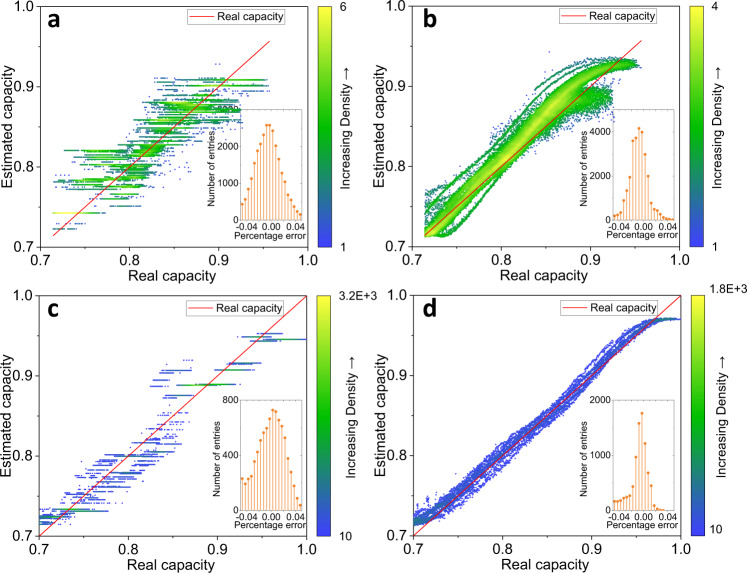
Test results of estimated capacity versus real capacity by transfer learning. The capacity results are uniformized by the nominal capacity for comparison. Results of TL2 embedding XGBoost method (**a**) and embedding SVR (**b**) on dataset 2. Results of TL2 embedding XGBoost method (**c**) and embedding SVR (**d**) on dataset 3. Additional results are disclosed in Supplementary Figs. 10–12.

The ZSL strategy obtains more than 3.4% test RMSE on all datasets directly using the base models. The error between the estimated capacity and real capacity is quite large as shown in Supplementary Fig. 10, meaning that the differences in battery types and materials cannot be ignored. When the base model is retrained in the No TL strategy, the XGBoost reaches a 2.9% test RMSE on dataset 2 and a 2.0% test RMSE on dataset 3, and the SVR gives no obvious improvement in the accuracy (Supplementary Fig. 11 and Supplementary Table 15). When the TL1 is applied on dataset 2 and dataset 3, the test RMSE of the SVR method goes down to 2.6 and 3.5% respectively, but a high number of outliers still appears in Supplementary Fig. 12. The results of estimated capacity versus real capacity by TL2 are presented in Fig. 6. The test RMSE is reduced to 2.4% by the XGBoost using the TL2 on dataset 2, noting that the performance of XGBoost using the No TL on dataset 3 is better than that of TL, which could be ascribed to the narrow distribution of capacity fade in dataset 3. The best accuracies on dataset 2 and dataset 3 are all reached by SVR using the TL2, showing test RMSEs of 1.7 and 1.6%, respectively. It can be concluded that the use of TL2 improves the estimation accuracy, and the reason behind the accuracy improvement is that a linear transformation of the input features helps the model adapt to the differences in battery types but similarity degradation modes. Interestingly, we find that the SVR is more reliable and suitable for transfer learning than the XGBoost with a small amount of newly collected data. The possible reason is that the XGBoost is a discrete gradient boosting framework, the output of the model is limited by the base model even if a new layer is added before the base model, whereas the SVR is a kernel-based framework, in which the continuous calculation achieves a better prediction under the designed TL2. In summary, the proposed approach using the relaxation voltage curve is useful to estimate the battery capacity, and the transfer learning improves the accuracy of capacity estimation requiring little tuning to adapt to the difference in batteries.

## Discussion

Accurate identification of lithium-ion battery capacity facilitates the accurate estimation of the driving range which is a primary concern for EVs. An approach without requiring information from the previous cycling to estimate battery capacity is proposed. The proposed approach uses three statistical features ([Var, Ske, Max]) extracted from the voltage relaxation curve as input to predict the capacity in the next cycle. The transfer learning embedding machine learning methods is applied on 130 cells to establish a suitable model and for the verification of the approach. The best base model achieves a root-mean-square error of 1.1%. The transfer learning adding a linear transformation layer before the base model shows good predictive ability within a RMSE of 1.7% on different batteries. The retraining of transfer learning only needs a small number of data units on the condition that a variation of the input data needs to be guaranteed to improve the applicability of the proposed approach. The relaxation process of a battery after full charge is easily obtained without the requirement of specific working conditions and voltage ranges, providing a new possibility for battery capacity estimation using data-driven methods in the system implementation of EV applications.

## Methods

### Cell selection and cycling

Commercially available lithium-ion batteries, i.e., LG INR18650-35E (3.5 Ah, 3.6 V), Samsung INR18650-MJ1 (3.5 Ah, 3.6 V), and Samsung INR18650-25R (2.5Ah, 3.6 V), have been tested. More battery specifications are listed in Supplementary Table 4. The positive electrode compositions of the INR18650-35E battery and INR18650-MJ1 battery are LiNi_0.86_Co_0.11_Al_0.03_O_2_ and Li(Ni_0.83_Co_0.11_Mn_0.07_)O_2_ respectively, and the negative electrodes for both cell types have roughly 97 wt% C and 2 wt% Si as well as traces of H, N, and S from Sorensen et al.^42^. The positive electrode of the INR18650-25R battery is the blend of 42 (3) wt.% Li(NiCoMn)O_2_ - 58 (3) wt.% Li(NiCoAl)O_2_, and the negative electrode is graphite from ref. ^18^. The INR18650-35E battery is named as NCA battery. The INR18650-MJ1 is named as NCM battery. The INR18650-25R is named as NCM + NCA battery according to the positive electrode. A potentiostat (BioLogic BCS-815, France) is employed for cell cycling. The measurements are conducted at 25, 35, and 45 °C in a climate chamber (BINDER, ±0.2 °C, Germany). Long-term cycling is conducted on a total of 130 cells with a summary of cycling conditions as provided in Table 1. A schematic connection of the potentiostat, chamber, and cells is shown in Supplementary Figure 13. For the NCA and NCM batteries, the metal taps are spot-welded to the cells, and the contact is soldered to the metal taps. A four-wire holder is used for the NCM + NCA battery. For partially charged/discharged NCA and NCM cells, the electrochemical impedance is measured in the fully charged state using a frequency range of 10 kHz to 50 mHz (20 data points per decade of frequency) and a potential amplitude of 20 mV. 30 min are set at the open circuit voltage before the electrochemical impedance tests. The electrochemical impedance is tested every 25 cycles for the NCA battery and every 50 cycles for the NCM battery. For the NCM + NCA battery, the electrochemical impedance is conducted every 50 cycles at full charge in a range of 10 kHz to 0.01 Hz (6 data points per decade of frequency) with a sinusoidal amplitude of 250 mA. 60 min are set at the open circuit voltage before the electrochemical impedance tests. The NCA cells and NCM cells are tested from 2016 to 2018, and the NCM + NCA cells are cycled in 2020. Different experimenters at different test periods are responsible for the difference in battery connection methods and experimental parameters in AC impedance tests, e.g., perturbation modes, perturbation amplitudes, and open circuit voltage time.

### Machine learning methods

Two transfer learning strategies embedding the XGBoost method and SVR method are applied in our study, and an illustration of the implemented transfer learning process is shown in Supplementary Fig. 9. The algorithms of the ElasticNet method, XGBoost method, and SVR method are introduced in Supplementary Note 5.

1. The base model is trained on all experimental data of NCA batteries (dataset 1). Firstly, the base model is directly verified on dataset 2 and dataset 3 without changing model weights as a zero-shot learning (ZSL) reference.
2. The base model is retrained using some new data units (Strategy D in Supplementary Note 4) as input variables from dataset 2 and dataset 3 as a No TL comparison.
3. Two transfer learning strategies (TL1 and TL2) are proposed by adding layers behind and in front of the base model. All weights in the base model are frozen in the transfer learning strategies except the newly added layer. In detail, TL1 means that a linear transformation layer is added before the output of capacity, which is described as

1 $${{{{{\rm{Q}}}}}}^{\prime} ={{{{{\rm{wQ}}}}}}+{{{{{\rm{b}}}}}}$$

TL2 means that a linear transformation layer is constructed to adapt the input features, which is described as

2 $$\left[\begin{array}{c}{{{{{\rm{Var}}}}}}^{\prime} \\ {{{{{\rm{Ske}}}}}}^{\prime} \\ {{{{{\rm{Max}}}}}}^{\prime} \end{array}\right]=W\left[\begin{array}{c}{{{{{\rm{Var}}}}}}\\ {{{{{\rm{Ske}}}}}}\\ {{{{{\rm{Max}}}}}}\end{array}\right]+b$$

*w, W*, and *b* are the weights in the added layer. The target dataset from dataset 2 and dataset 3 are selected to train the new layer weights.

The transfer learning models are verified on the remaining dataset 2 and dataset 3 respectively. The test RMSEs are compared in Table 3, and the estimation results are presented in Fig. 6 and Supplementary Figs. 10–12 for visualization purposes.

## Supplementary information

Supplementary Information

## Data Availability

The data generated in this study have been deposited in the Zenodo database under accession code [10.5281/zenodo.6379165].

## Code availability

The data processing is performed in python and is available at [https://github.com/Yixiu-Wang/data-driven-capacity-estimation-from-voltage-relaxation]. Code for the modeling work is available from the corresponding authors upon request.
