# Supplementary material for: Data-driven capacity estimation of commercial lithium-ion batteries from voltage relaxation
Source: Nat Commun. 2022 Apr 27;13:2261. doi: 10.1038/s41467-022-29837-w (PMC9046220; doi:10.1038/s41467-022-29837-w)
Supplement: Supplementary file 1 — Supplementary Information [file 41467_2022_29837_MOESM1_ESM.pdf]

Supplementary Information for
Data-driven capacity estimation of commercial lithium-ion batteries from voltage relaxation

Jiangong Zhu [1, 2, #], Yixiu Wang [3, #], Yuan Huang [1, 2], R. Bhushan Gopaluni [3], Yankai Cao [3], Michael Heere [2, 4], Martin J. Mühlbauer [2], Liuda Mereacre [2], Haifeng Dai [1 *], Xinhua Liu [5], Anatoliy Senyshyn [6], Xuezhe Wei [1], Michael Knapp [2 *], Helmut Ehrenberg [2]

[1] Clean Energy Automotive Engineering Center, School of Automotive Engineering, Tongji University, 201804 Shanghai, China

[2] Institute for Applied Materials (IAM), Karlsruhe Institute of Technology (KIT), 76344 Eggenstein-Leopoldshafen, Germany

[3] Department of Chemical and Biological Engineering, University of British Columbia, BC V6T 1Z3, Canada

[4] Technische Universität Braunschweig, Institute of Internal Combustion Engines, Hermann-Blenk-Straße 42, 38108 Braunschweig, Germany

[5] School of Transportation Science and Engineering, 100083, Beihang University, Beijing, China

[6] Heinz Maier-Leibnitz Zentrum (MLZ), Technische Universität München, Lichtenbergstr. 1, 85748 Garching b. München, Germany

[#] These authors contribute equally;

* Corresponding authors. Haifeng Dai (tongjidai@tongji.edu.cn), Michael Knapp (michael.knapp@kit.edu).

Supplementary Note 1: Analysis of real-time electric vehicle (EV) charging

A list of some EV charging strategies is summarized in Supplementary Table 2. The multistage current charge dividing the entire charging period into several charging stages is mainly applied in the EVs. As most of these charging data are held by some large private EV charging operators and EV manufacturers, it is hard to access from the public [1]. Thus, in-house real-time EV battery charging data from the China Automobile Academy of Engineering Co., Ltd. are collected and analyzed. Ten real-world EVs are randomly selected as shown in Supplementary Table 3. Six vehicles load the $Li(TM)O_2/C$ (TM = transition metal, $Li(TM)O_2$ is the positive electrode) batteries, and the other four vehicles use $LiFePO_4/C$ ($LiFePO_4$ is the positive electrode) batteries. Nine vehicles (1 ~ 9) use the multistage current charge method. Only Vehicle 10 is in constant current charge mode. The summary of the charging information is given in Supplementary Figure 1 and Figure 2. Three representative charging curves from vehicle 1, vehicle 9, and vehicle 10 are shown in Supplementary Figure 1 to illustrate the difference in charging protocols. We have counted the charging data of vehicle 1 and vehicle 10. The depth of charge during the vehicle operation is compared in Supplementary Figure 2. By ranking the charging start and end positions in ascending order, we find that the charging start position has no obvious distribution characteristics showing certain randomness, but the probability of being fully charged is high from the comparison of Supplementary Figure 2b (2e) and Supplementary Figure 2c (2f).

Supplementary Note 2: Discussion of data splitting methods on the base model

Four splitting strategies (A, B, C, and D) are compared, and the results are exhibited as follows. The training dataset and test dataset are in a 4:1 ratio, and 5-fold cross-validation is used to determine the hyperparameters of the model in the training process.

A. Temperature dependence splitting: The train and test results using the temperature-based data splitting method is shown in Supplementary Table 6, only the training on cells cycled at 25 °C and 45 °C, test on cells cycled 35 °C show a better estimation result.

B. Splitting on the time-series data: We use the former 80% data to train the model and use the later 20% data for the model test. The test accuracy is above 2.3% RMSE, indicating that it is difficult to estimate the model if the capacity decay is missing during the training process. The method cannot predict the battery future because battery degradation shows a nonlinear trend.

C. Random splitting method: All data are put together for random sampling without distinguishing the working conditions. Considering the variation of the data units under different cycling conditions, the weighted average method achieves similar estimation accuracy, presenting the effectiveness of the random data splitting method without data balance.

D. Cell stratified sampling on the working conditions: A stratified sampling method is used to select the cells in each working condition, meaning that the data from the same cell is either in the training set or in the test set. The cell splitting is approx. 4:1 for training and testing as presented in Supplementary Table 9. The result is quite good reaching 1.1% for the XGBoost and SVR methods.

By comparing the above data splitting methods, we find that the random splitting and the cell stratified sampling methods show similar and good test RMSEs. The temperature dependence splitting method is the worst. One possible reason is the unreasonable splitting of data amount between train and test data, for example, the ratio of data amount is 2:5 around (25 °C and 35 °C for training and 45 °C for testing). Another reason is that the machine learning algorithm is highly affected by working conditions, i.e., the model does not have the ability of "zero-shot learning" in the absence of the working condition in the training process. The time-series-based splitting method is also not ideal, meaning that a full range degradation dataset is necessary to train the model. This is due to the nonlinear attenuation of the battery and the strong influence of the working condition on battery degradation factors.

Supplementary Note 3: Description of benchmarking methods in Table 2

(1) Rest voltage based – Linear model:

The presented method in Ref. [2] is to study the change of U_{relax} with battery degradation. U_{relax} is defined as the open-circuit voltage of the battery after 30 min of

rest after full charging. The relationship between U_{relax} and capacity in dataset 1 is shown in Supplementary Figure 4. A linear model is trained based on a randomly selected 80% of the target dataset, and the remaining 20% of the data are used to test the model performance. The result shows that the proposed linear model achieves a RMSE of 2.5%.

(2) CC charge voltage based – RFR:

The presented method is based on the dependency of the battery capacity on the features extracted from the partial CC charge curve. The selected voltage range from 3.6 V to 3.8 V is selected according to Ref. [3], which is also corresponding to the middle SOC range in our work. For each charge curve from 3.6 V to 3.8 V in dataset 1, an interval of 2 mV is used to discretize it into 101 data points (V_0, V_1, …, V_k …, V_{100}). The features extracted as input are the relative capacity values Q_k (k = 0, 1, …, 100) at each voltage point, where Q_k is calculated based on coulomb counting by integrating the current with the time that the battery charged from V_0 to V_k. The initial capacity Q_0 is defined as 0. An example of feature extraction for one voltage curve is shown in Supplementary Figure 5. A random forest regressor is trained to map the sequence of the relative capacity values Q_k and battery capacity based on the randomly selected 80% dataset. For the hyperparameters of the random forest regression, the number of trees is chosen as 6 to be consistent with XGBoost and the number of random features for each split is chosen to be one-third of the number of variables. The prediction result on the remaining 20% dataset shows that the proposed method achieves a RMSE of 1.0%.

(3) ICA transformation – Linear model:

The method in Ref. [4] is to estimate the battery capacity based on remaining charge electricity (RCE), which is obtained by incremental capacity analysis (ICA) on battery charging voltage. A threshold is set according to the ICA value. Specifically, An ICA curve of one cell in dataset 1 is illustrated in Supplementary Figure 6a, in which the dashed line (dQ/dV = 2.5 mAh/mV) is defined as the threshold. The partial charged capacity from the threshold till to the end of the charge is counted as RCE. The relationship between RCE and battery capacity for all cells in dataset 1 is shown in Supplementary Figure 6b. A linear model is trained on the randomly selected 80% data samples, and the prediction performance on the remaining 20% samples shows that the proposed model achieves a RMSE of 1.3%.

(4) CC-CV charge voltage based – GPR:

The presented method in Ref. [5] estimates battery capacity using four specific features (F1, F2, F3, and F4) extracted from the CC-CV charge curve as shown in Supplementary Figure 7. F1 is the time of CC model duration, F2 is the time of CV mode duration, F3 is the slope of the curve at the end of CC charge mode and F4 is the vertical slope at the corner of the cc charge curve. A Gaussian process regression model with radial basis function (RBF) kernel and white noise kernel is trained based on the 80% dataset in dataset 1 and the remaining 20% dataset is used for model testing. The result shows that the proposed model achieves a RMSE of 1.1%.

Supplementary Note 4: Discussion of data selection strategies for the transfer learning retraining

For the transfer learning on dataset 2 and dataset 3, several data selection strategies (A, B, C, and D) on the TL2 are used and the results are compared in Supplementary Table 13.

A. Data selection according to the time-series data: 1% data are used in A1, and 10% data are used in A2. The RMSEs for the TL2 are quite large, illustrating the inappropriateness of the time-series-based splitting method in our study.

B. 1% random data: 1% of the target dataset from dataset 2 and dataset 3 are randomly set as the input variable to train the transfer learning model. 1.3% and 1.6% RMSE are obtained by SVR on dataset 2 and dataset 3 respectively.

C. Random cells from each working condition: A cell is randomly selected from each cycling condition, meaning that the data of three random cells corresponding to temperatures from dataset 2 and the data of three random cells corresponding to discharge rates from dataset 3 are used. A 1.4% RSME is achieved on dataset 2, and 1.6% RMSE is obtained on dataset 3. It is noted that the amount of input variable is approximately 7% for dataset 2 and 33% for dataset 3. Thus, a reduction of data volume is performed in Strategy D for a fairer comparison.

D. Reduction of data volume from the randomly selected cells. The data volume for the TL model re-training is reduced according to the battery cycle numbers. Data units are chosen from a randomly selected cell (as presented in Strategy **C**) with an interval of 100 cycles as the input variables. The sizes of the selected data units are summarized in Supplementary Table 14. It can be seen that the TL2 shows 1.7% RMSE on dataset 2, and 1.6% RMSE on dataset 3, respectively, proving the effectiveness of the transfer learning.

In a summary, we find that the model using strategies B, C, and D achieves good estimation accuracy. We speculate that these data selection methods contain the effect of working conditions, meaning that the diversity of working conditions is important to improve the model generalization as more input of the working conditions projects to more battery degradation pathways. The discussion of data splitting methods on the base model and transfer learning model proves that the generalization of the model is highly related to the working conditions of the battery.

Supplementary Note 5: Introduction of machine learning methods

ElasticNet method

The ElasticNet algorithm is proposed by Zou et al. [6], which is a regularized regression method that linearly combines the L_1 and L_2 penalties of the lasso and ridge methods. ElasticNet is an extension of ordinary least square (OLS) regression. In OLS regression, given d features, x_{i1}, …, x_{id}, the response y_i is predicted by:

$$\hat{y}_i=\beta_0+\sum_{j=1}^{d}\beta_j x_{ij} \tag{1}$$

A model fitting procedure produces the parameter vector $\hat{\beta}=(\hat{\beta}_0, \ldots, \hat{\beta}_d)$.

For the data set having n observations with p features, let $y=(y_1, \ldots, y_n)^T$, $X=\begin{bmatrix} x_{11} & \cdots & x_{1d} \\ \cdots & x_{ij} & \cdots \\ x_{n1} & \cdots & x_{nd} \end{bmatrix}$.

The ElasticNet loss function is defined as:

$$L(\lambda_1, \lambda_2, \beta)=\|y-X\beta\|_2^2+\lambda_2\|\beta\|_2^2+\lambda_1\|\beta\|_1 \tag{2}$$

If we set $\alpha=\lambda_2/(\lambda_1+\lambda_2)$, the optimized parameters vector is obtained by:

$$\hat{\beta}=\mathrm{argmin}_{\beta}L(\alpha,\beta)=\|y-X\beta\|_2^2+\alpha\|\beta\|_2^2+(1-\alpha)\|\beta\|_1 \tag{3}$$

where $\alpha\|\beta\|_2^2+(1-\alpha)\|\beta\|_1$ is called the ElasticNet penalty, which is a convex combination of the lasso and ridge penalty.

XGBoost method

The XGBoost method [7] is a scalable end-to-end tree boosting system designed to be highly efficient, flexible, and portable. It implements machine learning algorithms in the Gradient Boosting framework. Compared with multiple linear regression, XGBoost has the advantage of being able to handle nonlinear relationships. The tree $f(x)$ is defined as:

$$f_t(x)=\omega_{q(x)},\left(q\colon R^d\rightarrow\{1,2,\ldots,T\}, \omega\in R^T\right) \tag{4}$$

where t represents a tree, q represents the structure of each tree that maps an example to the corresponding leaf index. T is the number of leaves in the tree. Each f_t corresponds to an independent tree structure q and leaf weights ω (output of a tree).

The objective function is defined as:

$$obj^{(t)}=\sum_{i=1}^{n} l\left(y_i,\hat{y}_i^{(t)}\right)+\sum_{i=1}^{t}\Omega\left(f_i\right) \tag{5}$$

where l is a differentiable convex loss function that measures the difference between the prediction $\hat{y}$ and the target y_i. The second term Ω penalizes the complexity of the model, which helps to smooth the final learned weights to avoid over-fitting.

$$\Omega(f)=\gamma T+\frac{1}{2}\lambda\sum_{j=1}^{T}\omega_j^2 \tag{6}$$

where ω_j is the weight of the jth leaf node. γ and λ are the coefficients for penalty term Ω.

Using the second-order Taylor's formula, the objective function can be given as:

$$obj^{(t)}=\sum_{i=1}^{n} l\left(y_i,\hat{y}_i^{(t-1)}+f_t(x_i)\right)+\Omega(f_t)+\text{constant}$$
$$\approx\sum_{i=1}^{n}\left(l(y_i,\hat{y}_i^{(t-1)})+g_i f_t(x_i)+\frac{1}{2}h_i f_t^2(x_i)\right)+\Omega(f_t)+\text{constant} \quad (7)$$

where x_i is the input of the sample, $g_i=\partial_{\hat{y}^{(t-1)}}l(y_i,\hat{y}^{(t-1)})$ and $h_i=\partial^2_{\hat{y}^{(t-1)}}l(y_i,\hat{y}^{(t-1)})$

After removing the constant, the objective function at step t becomes

$$obj^{(t)}\approx\sum_{i=1}^{n}\left(g_i\omega_{q(x_i)}+\frac{1}{2}h_i\omega_{q(x_i)}^2\right)+\gamma T+\frac{1}{2}\lambda\sum_{j=1}^{T}\omega_j^2$$
$$=\sum_{j=1}^{T}\left(G_j\omega_j+\frac{1}{2}(H_j+\lambda)\omega_j^2\right)+\gamma T \quad (8)$$

where $G_j=\sum_{i\in I_j}g_i, H_j=\sum_{i\in I_j}h_i, I_j=\{i|q(x_i=j)\}$ is instance set of leaf j.

The optimal weight ω_j^* of leaf j for a fixed structure $q(x)$ can be computed by:

$$\omega_j^*=-\frac{G_j}{H_j+\lambda} \quad (9)$$

The optimal loss is:

$$obj^*=-\frac{1}{2}\sum_{j=1}^{T}\frac{(G_j)^2}{H_j+\lambda}+\gamma T \quad (10)$$

obj^* is a function of marking tree structure and measuring the quality of tree structure q. The smaller the value of obj^*, the better.

SVR method

SVR approach [8] is a kernel-based method that does not regress on the original input vector, but on its nonlinear expansion, which is mapped from a kernel function to a very high-dimensional feature space. Given a training set of data $\{(x_1, y_1),\dots,(x_n, y_n)\}$, where $x_i\subset R^d$ donates the input space of the sample, $y_i\subset R$ is the target value. $i=1,\dots,n$, corresponds to the size of the training data.

The generic SVR estimating function takes the form

$$\hat{y}_i=(\omega\cdot\Phi(x_i))+b \quad (11)$$

where $\omega\subset R^d, b\subset R$, and $\Phi(x)$ is a nonlinear transformation from R^d to a high-dimensional space. The ω has the following expansion:

$$\omega=\sum_{i=1}^{n}(\alpha_i-\alpha_i^*)\Phi(x_i) \quad (12)$$

where α_i and α_i^* are the Lagrange multiplier. With the expression of the kernel function $k(x_i,x)=\Phi(x_i)\cdot\Phi(x)$, the SVR estimating function can be expressed as:

$$\hat{y}_i=\sum_{i=1}^{n}\left(\alpha_i-\alpha_i^*\right)k(x_i,x)+b \tag{13}$$

The goal of SVR is to find the value of ω and b that minimizing the total loss

$$\min\left\{\frac{1}{2}\|\omega\|_2^2+C\sum_{i=1}^{l}l_\epsilon\left(\hat{y}_i-y_i\right)\right\} \tag{14}$$

where C is a constant, and vector l_ϵ is the loss function, the ϵ-insensitive loss function is used in this research:

$$l_\epsilon(\hat{y}_i-y_i)=\begin{cases}|\hat{y}_i-y_i|-\epsilon, & |\hat{y}_i-y_i|\geq\epsilon\\ 0, & |\hat{y}_i-y_i|<\epsilon\end{cases} \tag{15}$$

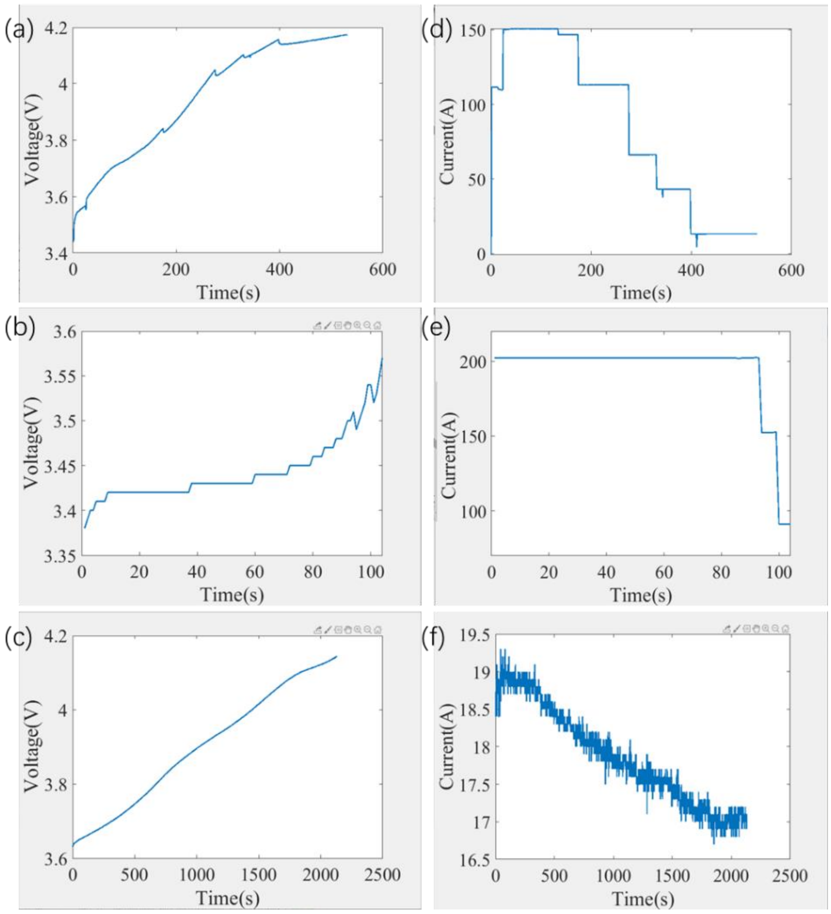

Supplementary Figure 1 Randomly selected battery charging data. Voltage profile (**a**) and current profile (**d**) from vehicle 1 with $Li(TM)O_2/C$ (TM = transition metal, $Li(TM)O_2$ is the positive electrode) battery, Voltage profile (**b**) and current profile (**e**) from vehicle 9 with $LiFePO_4/C$ ($LiFePO_4$ is the positive electrode) battery, Voltage profile (**c**) and current profile (**f**) from vehicles 10 ($Li(TM)O_2/C$).

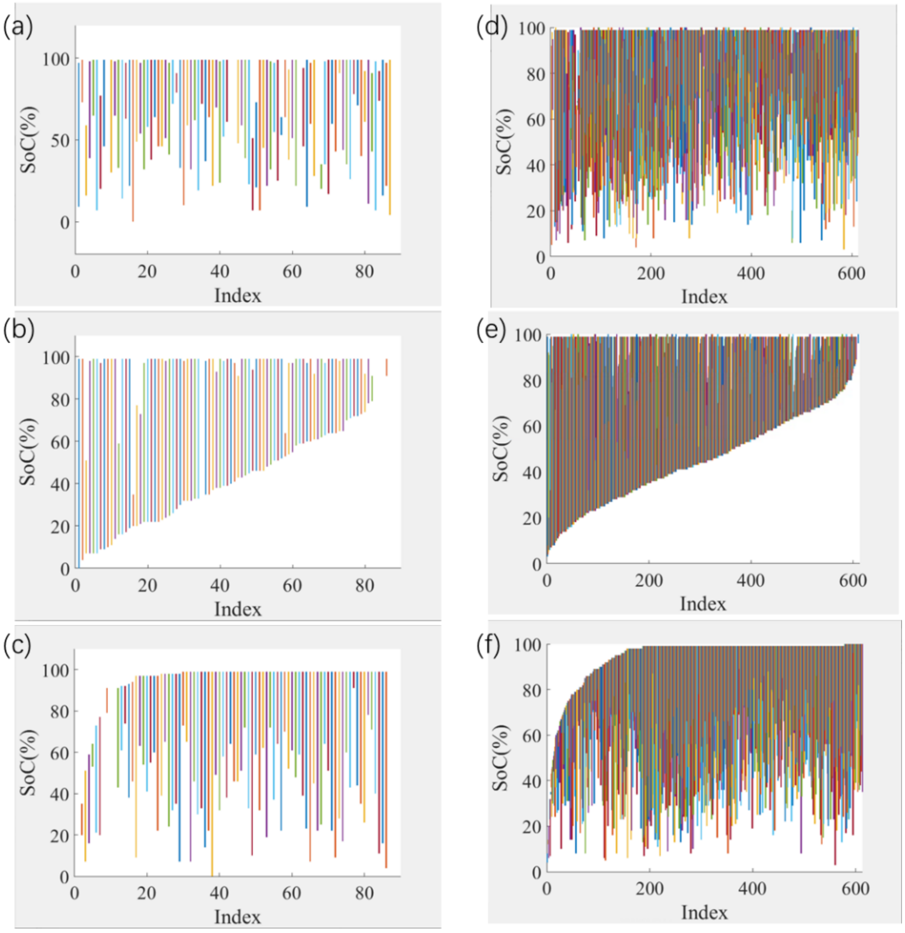

Supplementary Figure 2 The state of charge (SoC) according to the time sequence of vehicle 1 (**a**) and vehicle 10 (**d**). Start of charge point in ascending order of vehicle 1 (**b**) and vehicle 10 (**e**). End of charge point in ascending order of vehicle 1 (**c**) and vehicle 10 (**f**).

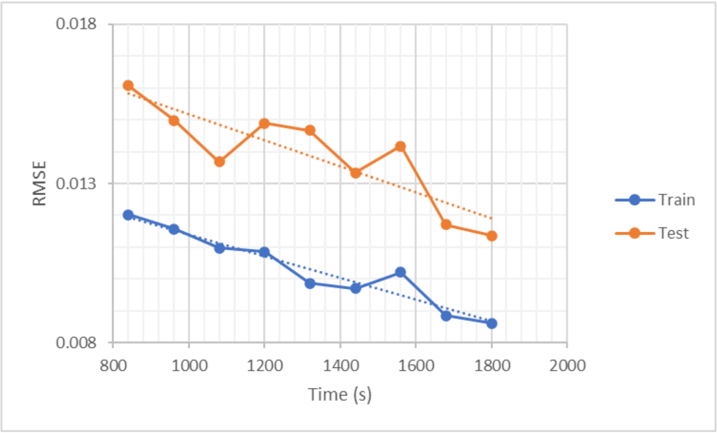

Supplementary Figure 3 The train and test root-mean-square error (RMSE) under different relaxation durations by the XGBoost method. The relaxation duration is divided into nine ranges, and it presents the RMSE decreases as the relaxation time increases, indicating that longer relaxation time improves the model accuracy. The

possible explanation is that the longer the relaxation time is, the relaxation voltage is close to the battery OCV (Open Circuit Voltage), which has been proved to be an important parameter to probe battery capacity.

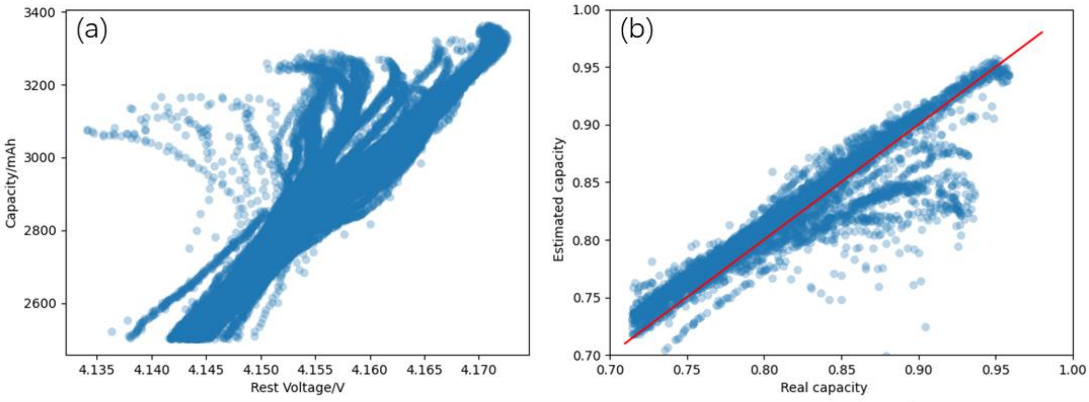

Supplementary Figure 4 Results of the Rest voltage based - Linear model. Features extraction (**a**) and test results of estimated capacity versus real capacity (**b**)

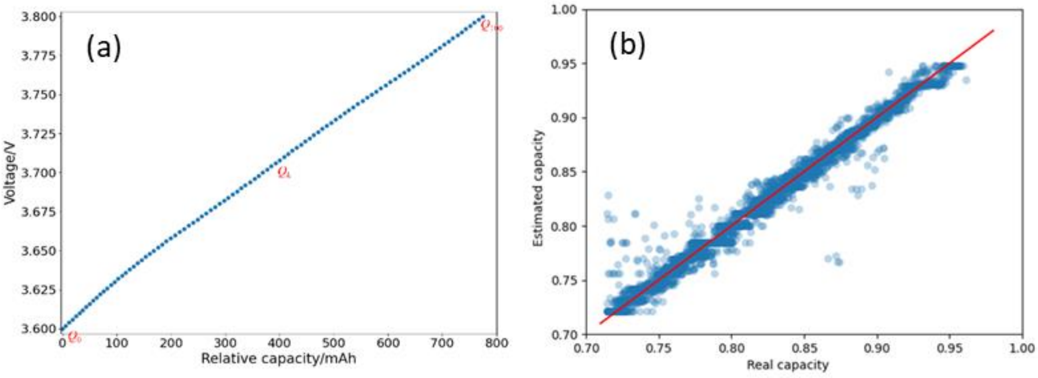

Supplementary Figure 5 Results of the CC charge voltage based – RFR. Features extraction (**a**) and test results of estimated capacity versus real capacity (**b**)

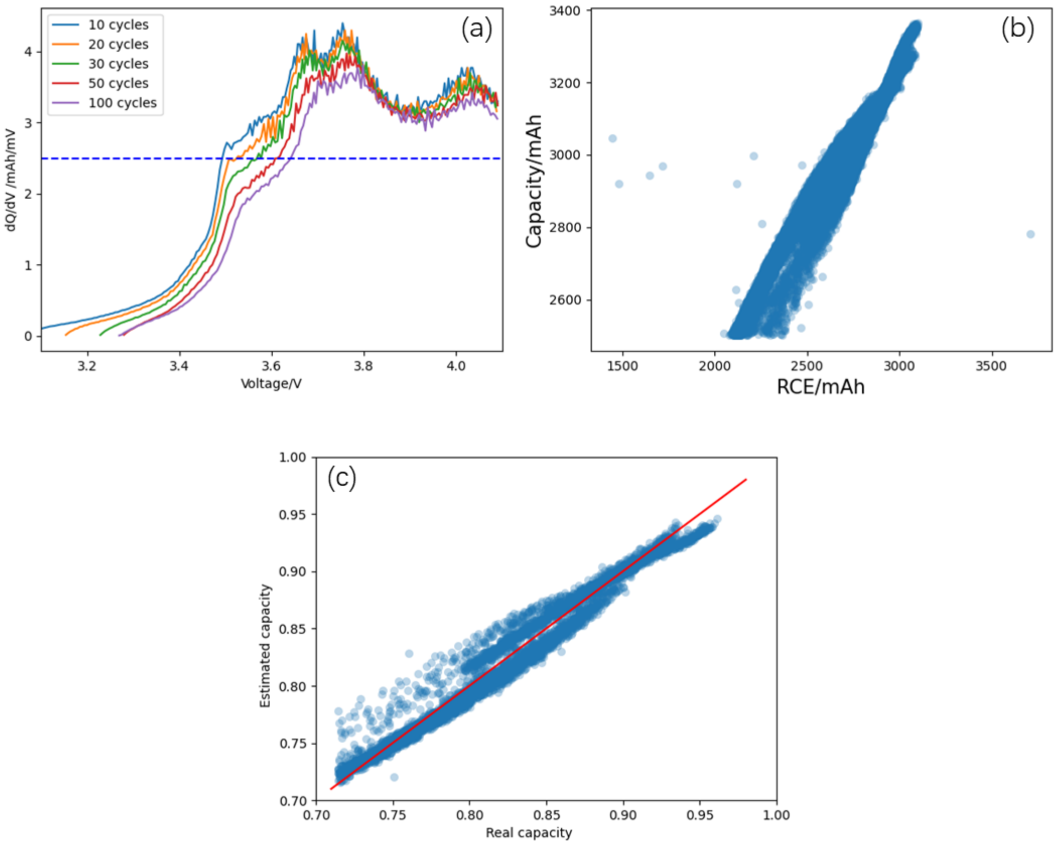

Supplementary Figure 6 Results of the ICA transformation – Linear model. Features extraction (**a**), RCE versus capacity (**b**), and test results of estimated capacity versus real capacity (**c**)

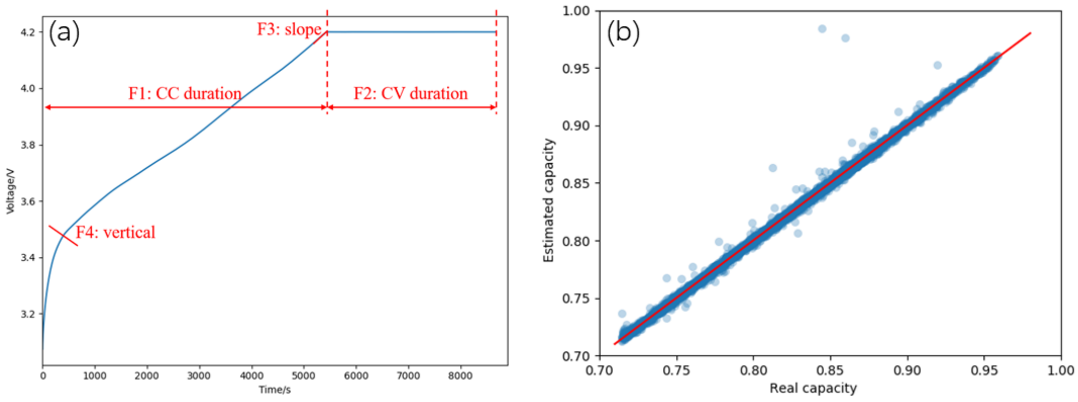

Supplementary Figure 7 Results of the CC-CV charge voltage-based method – GPR. Features extraction (**a**) and test results of estimated capacity versus real capacity (**b**)

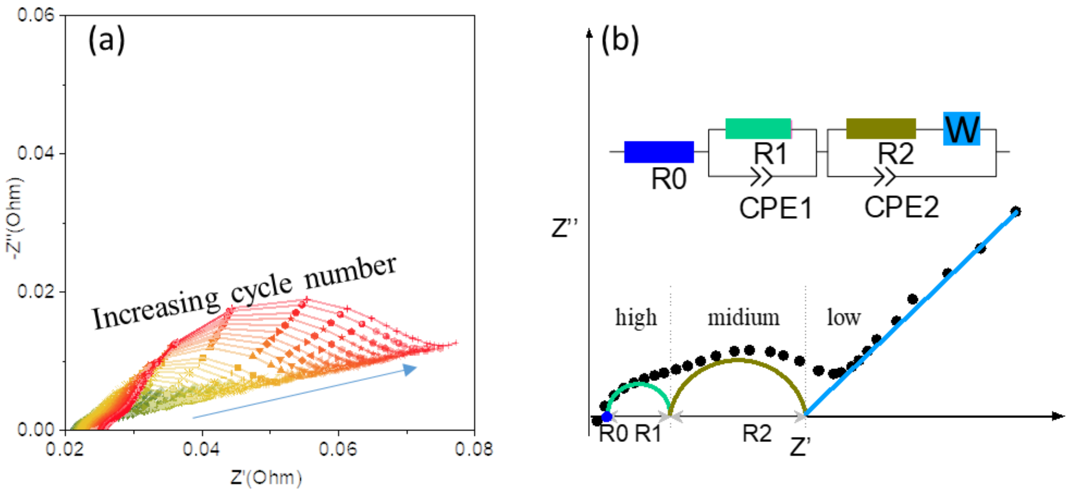

Supplementary Figure 8 A schematic plot of impedance spectra during cycling (**a**) and the corresponding equivalent circuit model (ECM) (**b**). The impedance spectra in **a** are all tested results using a "line +scatter" plots for visualization. R0 is the real part of the impedance at zero crossing. R1//CPE1 in parallel denotes the migration of lithium ions through the solid electrolyte interphase in the high frequency range. The semi-circle in the medium frequency range is accounted for the charge transfer process, and modeled as R2 in parallel with CPE2 (R2//CPE2). The low frequency slope is associated with Warburg impedance (W). The fitting coefficient of determination (R^2) between the raw and fitted impedance data is summarized in Supplementary Table 12. All the raw impedance data and fitted data are shared in the data availability (https://doi.org/10.5281/zenodo.6379165).

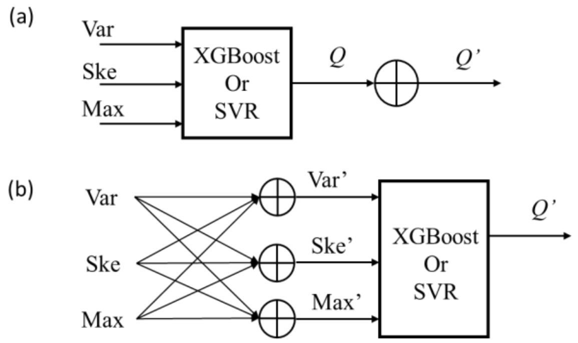

Supplementary Figure 9 Illustration of the implemented transfer learning process. TL1 (a) and TL2 (b). Variance (Var), skewness (Ske), and maxima (Max) are the input features. SVR means Support Vectors Regression.

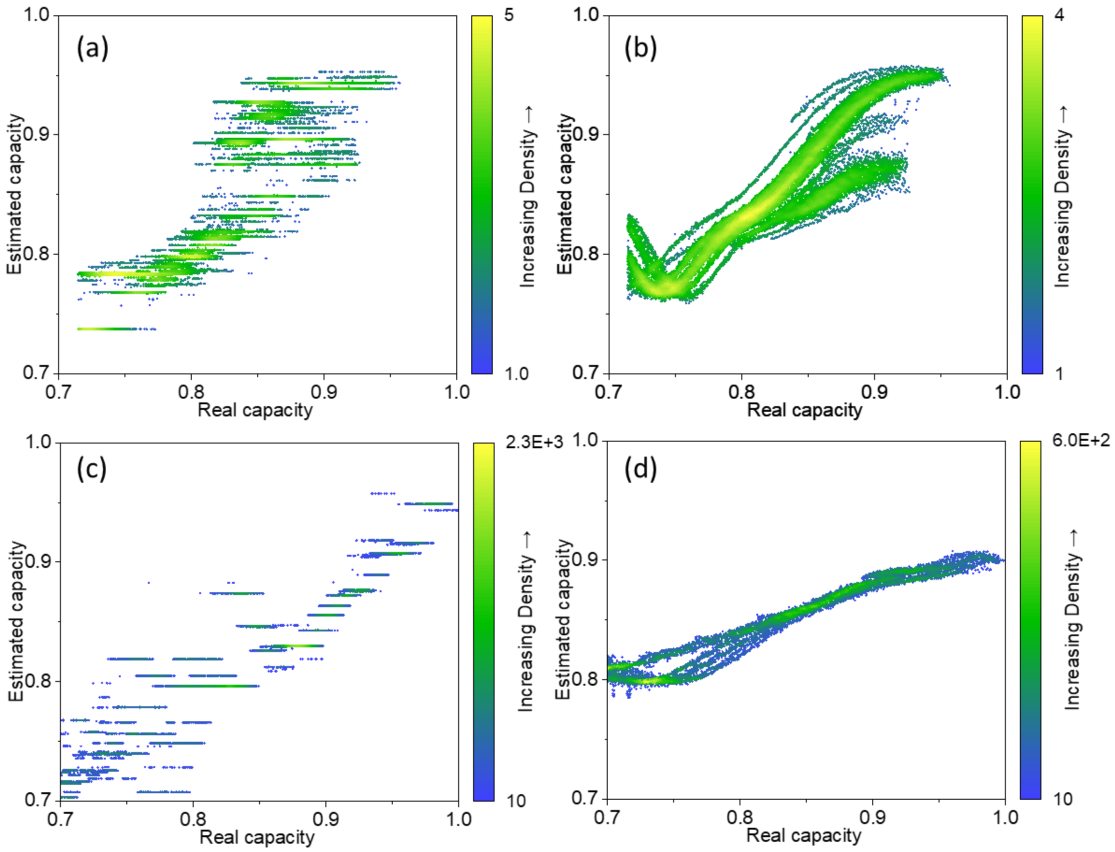

Supplementary Figure 10 Test results of estimated capacity versus real capacity by transfer learning. Results of ZSL embedding XGBoost method (**a**) and embedding SVR method (**b**) on dataset 2. Results of ZSL embedding XGBoost method (**c**) and embedding SVR method (**d**) on dataset 3.

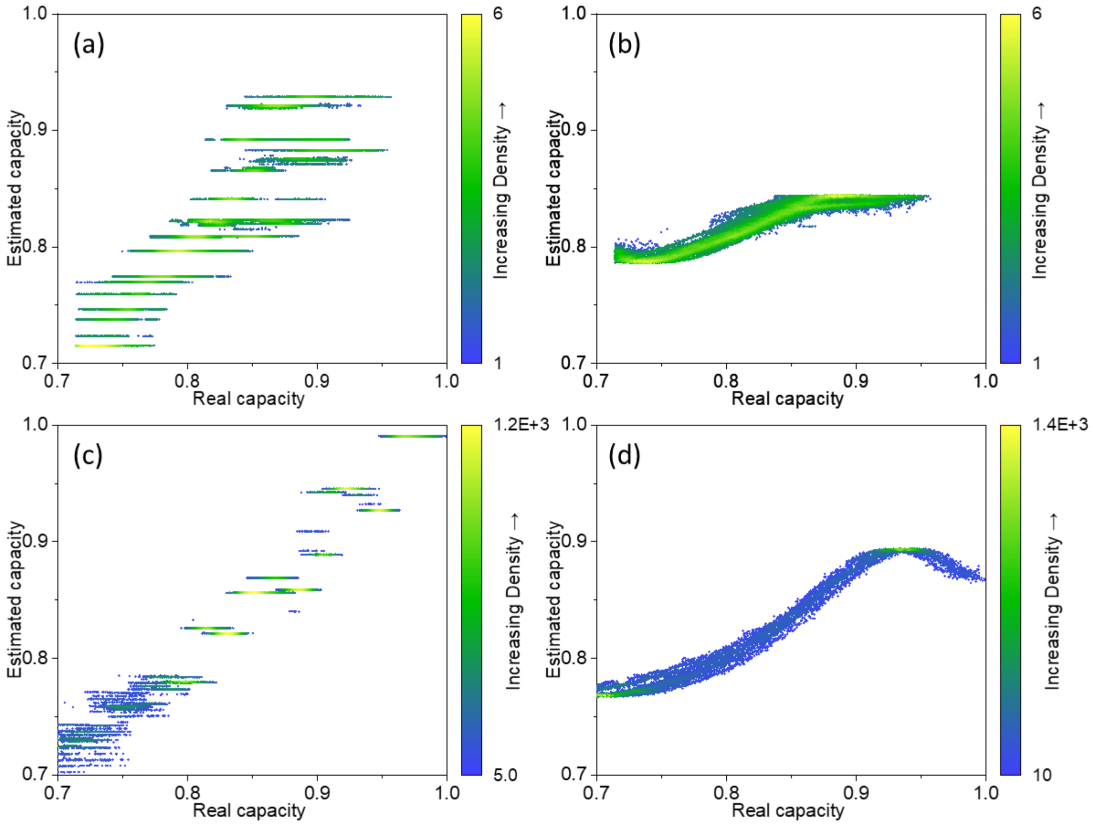

Supplementary Figure 11 Test results of estimated capacity versus real capacity by transfer learning. Results of No TL embedding XGBoost method (**a**) and embedding SVR method (**b**) on dataset 2. Results of No TL embedding XGBoost method (**c**) and embedding SVR method (**d**) on dataset 3. The poor performance of SVR on No TL is limited using the "radial based function" kernel in the SVR model. The performance of using a "linear" kernel instead of the "radial based function" kernel in the SVR model is better as shown in Supplementary Table 15, achieving a better estimation accuracy.

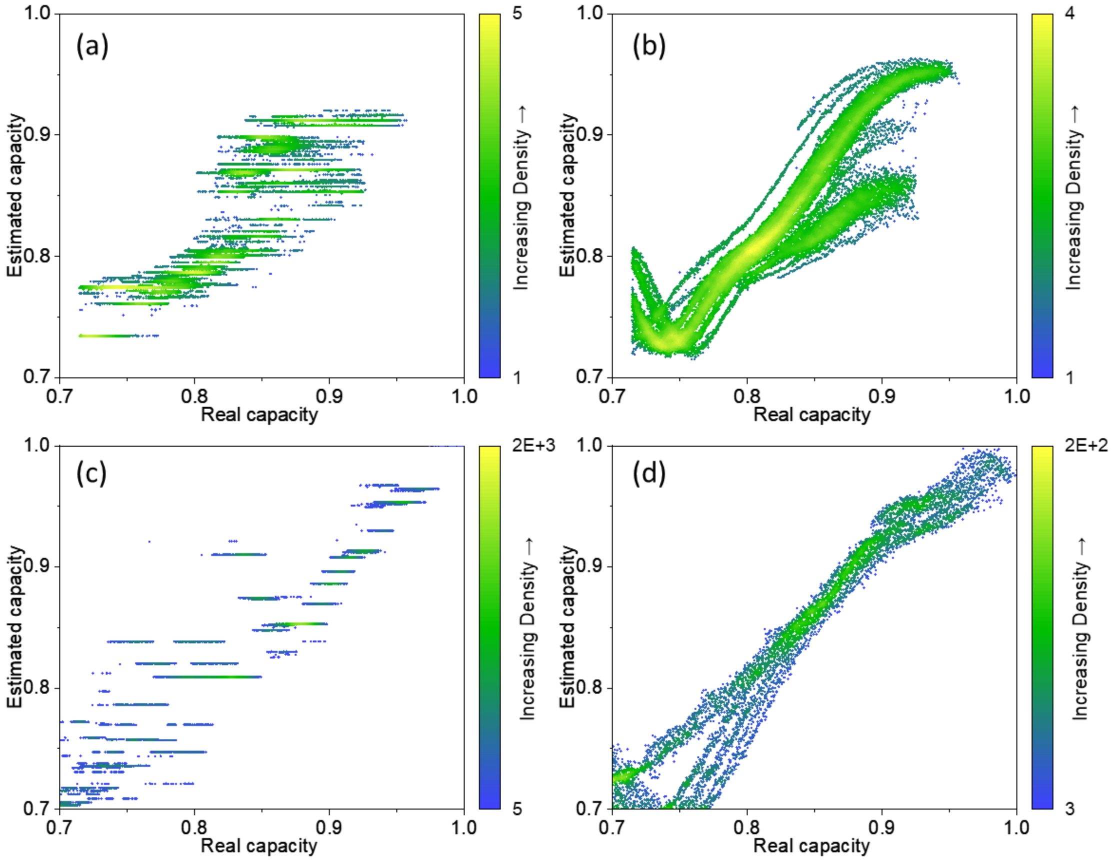

Supplementary Figure 12 Test results of estimated capacity versus real capacity by transfer learning. Results of TL1 embedding XGBoost method (**a**) and embedding SVR method (**b**) on dataset 2. Results of TL1 embedding XGBoost method (**c**) and embedding SVR method (**d**) on dataset 3.

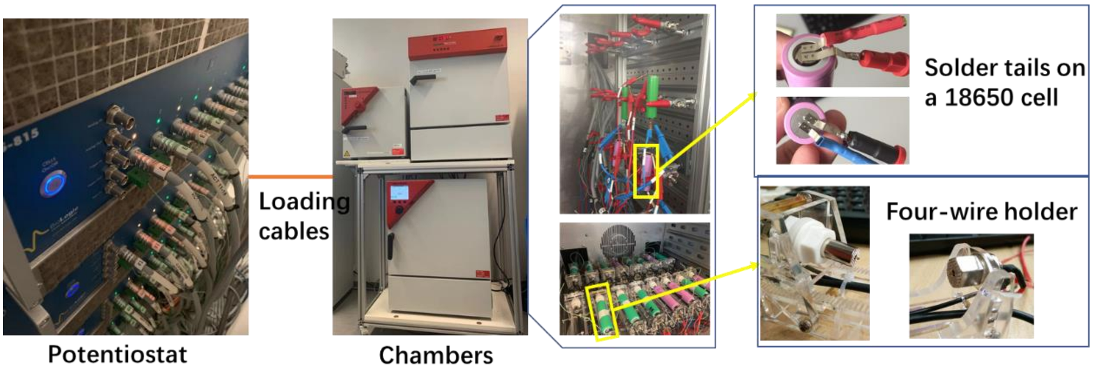

Supplementary Figure 13 The schematic connection of the potentiostat, chamber, and cells. For the NCA and NCM batteries, the metal taps are spot-welded to the cells, and the contact is soldered to the metal taps. A four-wire holder is used for the NCM+NCA battery.

Supplementary Table 1 A summary of the typical capacity estimation methods. It is noted that the table only lists the estimation accuracy of these methods on their specific

data (as marked as the "battery dataset" column). Because the performance of the machine learning model is dependent on the quality and quantity of input data, they cannot be compared directly unless using the same data.

Features from	Applied methods		Battery dataset	Estimation precision in each study	
(**I**) CC (Constant Current) charge voltage-based	PSO (particle swarm Optimization) [9]		Designed experiments at various temperatures, current rates, and SoC ranges on nine NCM+LMO/C cells, two NCM /C cells, and one LMO/C cell	Root-mean-square error (RMSE)	1.91%
	LSTM (long short-term memory network) +TL (transfer learning) [10]		Laboratory NCM batteries cycled at room temperature<br>CALCE data*<br>LFP data*	RMSE	0.59%
	GPR (Gaussian process regression) [11]		NASA data*<br>Oxford data*	RMSE	2%-3%
	LSTM-RNN (Recurrent neural network) [12]		In-house experiments on 48 NCM batteries using one specific cycling profile	Mean absolute percentage error (MAPE)	0.76%
	RFR (random forest regression) [3]		17 NCM batteries are cycled at different DoD and temperatures	RMSE	0.48%-1.3%
	dNNe (deep neural network ensemble) [13]		Oxford data*	RMSE	0.39%
	dNNe[13]		LFP data*	RMSE	0.45%
	ICA (Incremental Capacity Analysis) transformation [14]	SVR (support vectors regression) [15]	Two type NCM batteries (two NCM cells and three $LiFePO_4$ cells) are cycled at 24 °C using 3C and 4C discharge rates respectively	RMSE	<1.80%
		GPPF (Gaussian Process Particle Filter) [16]	CALCE data*	RMSE	0.82%-1.24%
		GPR[17]		RMSE	1.64%-4.73%
		Linear model[4]	NASA data*	RMSE	0.56%-1.25%
		BPNN (back-	Eight battery modules (13 cells in parallel in one module) in	RMSE	0.535%

		propagation neural network) [18]	series are cycled by 1C charge/discharge at 25±1°C		
		Power law and linear fitting [19]	Calendar aging of six LMO+NCM batteries at various SoC and temperatures	RMSE	2.99%
**(II)** CC-CV (Constant Current Constant Voltage) charge voltage-based	GPR [5]		NASA data*	RMSE	0.78%-3.45%
	RVM (Relevance Vector Machine) [20]		NASA data*	RMSE	1.37%-4.22%
	DNNe [13]		NASA data*	RMSE	4.26%
**(III)** Rest voltage-based	Linear model [2]		LMO+NMC/C, NCM, and LFP batteries are tested at different temperatures with calendar and cycling aging mode	percentage error	0.7%-3.3% (for LMO+NMC/C battery)
Other: Current in CV charge	Exponential law model [21]		NCA cells are stored under different temperatures and So (three cells in each condition)	R^2	0.9931

The data marked by the asterisk (*) are the publicly available datasets.
CALCE data*: https://web.calce.umd.edu/batteries/data.htm;
LFP data*: https://data.matr.io/1/projects/5c48dd2bc625d700019f3204;
NASA data*: https://ti.arc.nasa.gov/tech/dash/groups/pcoe/prognostic-data-repository/;
Oxford data*: https://ora.ox.ac.uk/objects/uuid:03ba4b01-cfed-46d3-9b1a-7d4a7bdf6fac;

Supplementary Table 2 A list of some EV charging strategies

EV type/ company name	Charging information	Charging type/ Power rate	Reference or link
*SAE J1772	4h to 14h (e.g. home charging)	Slow: <3.7 kW	[22,23]
Chevrolet Bolt EV	11.5 kW level 2 charging capability and standard DC fast charging capability	Semi-fast: 11.5 kW	https://www.chevrolet.com/electric/bolt-ev
BEIJING EU7	30%-80% SoC, 25 degree, fast charge in 30 minutes	Fast: 50-150 kW	https://eu7.beijingauto.com.cn/
Tesla Model S	Supercharge up to 200 miles in 15 minutes	Fast: 50-150 kW	https://www.tesla.com/models

Porsche Taycan	DC 50 kW, 5%-80% SoC in 93 minutes	Fast: 50-150 kW	https://cc.porsche.cn/icc/ccCall.do?rt=1641797730&screen=1280x720&userID=DE&lang=de&PARAM=parameter_internet_de&ORDERTYPE=Y1AAA1&CNR=C00&MODELYEAR=2022&hookURL=https%3a%2f%2fwww.porsche.com%2fgermany%2fmodelstart%2fall%2f
BMW i4	DC fast-charging capacity of 'up to 205 kW	Fast: up to 205 kW	https://electrek.co/2021/11/29/bmw-starts-i4-electric-car-deliveries-customers/
BMW iX xDrive50 (expected to arrive in 2022)	Recoup up to 90 miles of range in 10 minutes of DC fast charging	Fast: 50-150 kW probably	https://electrek.co/2022/01/10/22-of-the-most-anticipated-electric-vehicles-coming-in-2022/#h-bmw-ix-xdrive50
Lotus 'Type 132' SUV (expected to arrive in Spring of 2022)	Can charge to 80% charge in around 20 minutes on an 800V charger	Fast: 50-150 kW probably	https://electrek.co/2022/01/10/22-of-the-most-anticipated-electric-vehicles-coming-in-2022/#h-lotus-type-132-suv
XPeng G9 (aimed to start production in China in Q3 of 2022)	Can charge up to 200 km of range in 5 minutes	Fast: 50-150 kW probably	https://electrek.co/guides/gm/#h-xpeng-g9
Toyota bZ4X	Can charge to 80% SOC in about 30 minutes with 150 kW DC fast-charging capacity	Fast: 50-150 kW probably	https://electrek.co/2021/10/29/toyota-unveils-first-all-electric-car-bz4x-an-electric-suv-packed-cool-features/
BYD Tang EV	30%-80% SoC, fast charge in 0.5 hours	Fast: 50-150 kW	https://www.bydauto.com.cn/auto/carShow.html-param=2021%E6%AC%BE%E5%94%90EV
Porsche	Extreme fast charging	Extreme fast: >350kW	[24]

*SAE J1772: SAE Electric Vehicle and Plug in Hybrid Electric Vehicle Conductive Charge Coupler,

Standard SAE J1772, Oct. 2017, p. 1.

Supplementary Table 3 A summary of the charging mode of real-time EVs

Vehicle	Charging mode	Battery type
1	Multistage current charge	$Li(TM)O_2/C$, TM= transition metal, $Li(TM)O_2$ is the positive electrode.
2		
3		
4		
5		
6		$LiFePO_4/C$, $LiFePO_4$ is the positive electrode.
7		
8		
9		
10	Constant current charge	$Li(TM)O_2/C$, TM= transition metal, $Li(TM)O_2$ is the positive electrode.

Supplementary Table 4 Specifications of NCA, NCM, and NCM+NCA batteries

Sample name	NCA battery	NCM battery	NCM+NCA battery
Battery type	18650		
Anode material	Graphite/Si[25]		Graphite[26]
Cathode material	$Li_{0.86}Ni_{0.86}Co_{0.11}Al_{0.03}O_2$ (NCA) [25]	$Li_{0.84}(Ni_{0.83}Co_{0.11}Mn_{0.07})O_2$ (NCM) [25]	42 (3) wt.% $Li(NiCoMn)O_2$ blended with 58 (3) wt.% $Li(NiCoAl)O_2$ (NCM+NCA) [26]
Electrolyte	Non-aqueous solution with lithium hexafluorophosphate ($LiPF_6$)		
Nominal voltage	3.6 V		
Cutoff voltage	2.65 V - 4.2 V	2.5 V - 4.2 V	
Nominal capacity	3.5 Ah		2.5 Ah
Battery mass	45.0 g		

Supplementary Table 5 Statistical features extracted from one voltage relaxation curve, x_i is the battery terminal voltage, i=1, …, n, n is the number of samples in one relaxation curve

Maxima (Max)	$x_{\max} = \max\{x_i\}$
Mean (Mean)	$\bar{x} = \frac{1}{n}\sum_{i=1}^{n} x_i$
Minima (Min)	$x_{\min} = \min\{x_i\}$

Variance (Var)	$\mu_2 = \frac{1}{n-1}\sum_{i=1}^{n}(x_i - \bar{x})^2$
Skewness (Ske)	$\mu_3 = \frac{1}{n}\sum_{i=1}^{n}\left(\frac{x-\bar{x}}{\sqrt{\mu_2}}\right)^3$
Excess Kurtosis (Kur)	$\mu_4 = \frac{1}{n}\sum_{i=1}^{n}\left(\frac{x-\bar{x}}{\sqrt{\mu_2}}\right)^4 - 3$

Supplementary Table 6 Test results of temperature dependence splitting method

Train	Test	Dataset 1		Train: test
		XGBoost	Support Vectors Regression (SVR)	
25 °C, 35 °C	45 °C	0.025	0.031	2:5
25 °C, 45 °C	35 °C	0.015	0.015	19:1
35 °C, 45 °C	25 °C	0.044	0.038	3:1

Supplementary Table 7 Test results of splitting on the time-series data

Dataset 1	
XGBoost	0.023
Support Vectors Regression (SVR)	0.031

Supplementary Table 8 Test results of splitting on random splitting method

	Dataset 1	
	XGBoost	Support Vectors Regression (SVR)
Without data balance	0.011	0.011
With data balance (weighted average)	0.013	0.011

Supplementary Table 9 Test results of cell stratified sampling method

	Train (cells)	Test (cells)	Dataset 1	
			XGBoost	Support Vectors Regression (SVR)
CY25-0.25/1	6	1	0.011	0.011
CY25-0.5/1	15	4		
CY25-1/1	7	2		
CY35-0.5/1	2	1		

CY45-0.5/1	22	6		

Supplementary Table 10 The list of feature combinations (*i*, *j*) corresponding to Figure 3 [0,1,2,3,4,5] = [Var, Ske, Max, Min, Mean, Kur]

j	**8**	[1,3,5]	[0,1,2,3]	[0,1,2,4]	[0,1,2,5]	[0,1,2,3,4]	[0,1,2,3,5]	[1,2,3,4,5]	
	**7**	[1,2,5]	[1,3,4]	[3,4,5]	[0,1,3,4]	[0,1,3,5]	[2,3,4,5]	[0,2,3,4,5]	[0,1,2,3,4,5]
	**6**	[4,5]	[1,2,3]	[1,2,4]	[2,4,5]	[0,1,4,5]	[0,2,3,4]	[2,3,4,5]	[0,1,3,4,5]
	**5**	[3,4]	[3,5]	[0,3,5]	[0,4,5]	[2,3,5]	[0,2,3,5]	[0,2,4,5]	[0,1,2,4,5]
	**4**	[2,3]	[2,4]	[2,5]	[0,2,5]	[0,3,4]	[2,3,4]	[0,3,4,5]	[1,3,4,5]
	**3**	[5]	[1,3]	[1,4]	[1,5]	[0,2,3]	[0,2,4]	[1,4,5]	[1,2,3,5]
	**2**	[1]	[3]	[0,4]	[0,5]	[1,2]	[0,1,4]	[0,1,5]	[1,3,4,5]
	**1**	[0]	[2]	[4]	[0,1]	[0,2]	[0,3]	[0,1,2]	[0,1,3]
		**1**	**2**	**3**	**4**	**5**	**6**	**7**	**8**
		i							

Supplementary Table 11 Hyperparameters in ElasticNet, XGBoost, and Support Vector Regression (SVR)

ElasticNet		XGBoost		SVR	
alphas	{0.001+(1-0.001)/19*i|i=0..19}5	learning_rate	0.8	kernels	'rbf'
l1_ratio	{0.001+(1-0.001)/19*i|i=0..19}	N_estimators	6	epsilons	20
cv	5	Max_depth	6	Cs	20
		Objective	reg:squarederror	gammas	'auto'
		Lambda	1		
		alpha	0		
		subsample	1		

Supplementary Table 12 The coefficient of determination (R^2) between the raw and fitted impedance data. All the raw impedance data and fitted data are shared in the data availability (https://doi.org/10.5281/zenodo.6379165). The R^2 marked with * means that no R1 and CPE1 (shown in Supplementary Figure 8b) are used for the data fitting.

NCA battery					
Cycle	CY25-0.25/1	CY25-0.5/1	CY25-1/1	CY35-0.5/1	CY45-0.5/1
0	0.99993*	0.99990*	0.99999*	0.99986*	0.99981*
25	0.99990*	0.99997	0.99996*	0.99989*	0.99982*
50	0.99996	0.99880	0.99997*	0.99711*	0.99975*
75	0.99993	0.99997	0.99999	0.99998	0.99980
100	0.99994	0.99997		0.99988	0.99981
125	0.99992	0.99997		0.99988	0.99988
150	0.99992	0.99998		0.99988	0.99989

175	0.99996	0.99999		0.99989	0.99988
200	0.99994			0.99989	0.99982
225	0.99996			0.99989	0.99981
250	0.99996			0.99989	0.99982
275	0.99996			0.99990	0.99983
300	0.99994			0.99990	0.99987
325	0.99992			0.99990	0.99989
350	0.99995			0.99991	0.99988
375				0.99991	0.99990
400				0.99994	0.99989
425				0.99992	0.99990
450				0.99993	0.99989
475				0.99993	0.99989
500				0.99994	0.99991
525				0.99994	0.99991
550				0.99996	0.99991
575				0.99996	0.99993
600					0.99993
NCM battery					
Cycle	CY25-0.5/1	CY35-0.5/1	CY45-0.5/1		
0	0.99973	0.99994*	0.99934*		
50	0.99970	0.99992	0.99953		
100	0.99981	0.99950	0.99951		
150	0.99982	0.99964	0.99953		
200	0.99987	0.99977	0.99969		
250	0.99989	0.99975	0.99971		
300	0.99989	0.99976	0.99984		
350	0.99989	0.99980	0.99983		
400	0.99999	0.99976	0.99985		
450		0.99981	0.99987		
500		0.99979	0.99984		
550		0.99982	0.99985		
600		0.99980	0.99991		
650		0.99982	0.99989		
700		0.99986	0.99990		
750		0.99986			
800		0.99986			
850		0.99985			
900		0.99986			
950		0.99985			
1000		0.99986			
1050		0.99988			

1100		0.99987			
1150		0.99989			
1200		0.99989			
1250		0.99985			
1300		0.99989			
1350		0.99990			
NCM+NCA battery					
Cycle	CY25-0.5/1	CY25-0.5/2	CY25-0.5/4		
0	0.99977	0.99967	0.99976		
100	0.99988	0.99985	0.99974		
200	0.99987	0.99990	0.99983		
300	0.99997	0.99997	0.99991		
400	0.99997	0.99996	0.99988		
500	0.99995	0.99994	0.99984		
600	0.99994	0.99992	0.99970		
700	0.99992	0.99988	0.99991		
800	0.99983	0.99990	0.99991		
900	0.99995	0.99994	0.99993		
1000	0.99986	0.99994	0.99994		

Supplementary Table 13 Test results of different data selection methods used in the transfer learning (TL2)

	Data for TL retraining	Dataset 2 (TL2)		Dataset 3 (TL2)	
		XGBoost	SVR	XGBoost	SVR
A1	1% data on the time series data	0.041	0.050	0.038	0.070
A2	10% data on the time series data	0.028	0.037	0.073	0.070
B	1% random data	0.015	0.013	0.026	0.016
C	Random cell from each working condition	0.016	0.014	0.022	0.016
D	Reduced data volume (by cycles) from the random selected cells	0.024	0.017	0.024	0.016

Supplementary Table 14 Size comparison of data units in strategy D for transfer learning

		Source data units	data units for retaining
Dataset 2	CY25-0.5/1	5490	2
	CY35-0.5/1	4712	13
	CY45-0.5/1	17600	4

Dataset 3	CY25-0.5/1	2843	10
	CY25-0.5/2	2913	10
	CY25-0.5/4	2826	10

Supplementary Table 15 Test result of a “linear” kernel for the SVR model in No TL

Dataset 2	1.9%
Dataset 3	2.0%

Supplementary References

1 Jia, Q.-S. & Long, T. A review on charging behavior of electric vehicles: data, model, and control. *Control Theory and Technology* **18**, 217-230 (2020).

2 Baghdadi, I., Briat, O., Gyan, P. & Vinassa, J. M. State of health assessment for lithium batteries based on voltage–time relaxation measure. *Electrochim. Acta* **194**, 461-472, doi:10.1016/j.electacta.2016.02.109 (2016).

3 Li, Y. *et al.* Random forest regression for online capacity estimation of lithium-ion batteries. *Applied Energy* **232**, 197-210, doi:10.1016/j.apenergy.2018.09.182 (2018).

4 Pei, P. *et al.* Capacity estimation for lithium-ion battery using experimental feature interval approach. *Energy* **203**, doi:10.1016/j.energy.2020.117778 (2020).

5 Yang, D., Zhang, X., Pan, R., Wang, Y. & Chen, Z. A novel Gaussian process regression model for state-of-health estimation of lithium-ion battery using charging curve. *J. Power Sources* **384**, 387-395, doi:10.1016/j.jpowsour.2018.03.015 (2018).

6 Zou, H. & Hastie, T. Regularization and variable selection via the elastic net. *J. Roy. Stat. Soc. Ser. B. (Stat. Method.)* **67**, 301-320 (2005).

7 Chen, T. & Guestrin, C. in *Proceedings of the 22nd acm sigkdd international conference on knowledge discovery and data mining.* 785-794.

8 Awad, M. & Khanna, R. in *Efficient learning machines* 67-80 (Springer, 2015).

9 Zheng, Y. *et al.* A novel capacity estimation method based on charging curve sections for lithium-ion batteries in electric vehicles. *Energy* **185**, 361-371, doi:10.1016/j.energy.2019.07.059 (2019).

10 Shu, X. *et al.* A Flexible State-of-Health Prediction Scheme for Lithium-Ion Battery Packs With Long Short-Term Memory Network and Transfer Learning. *IEEE Transactions on Transportation Electrification* **7**, 2238-2248, doi:10.1109/tte.2021.3074638 (2021).

11 Richardson, R. R., Birkl, C. R., Osborne, M. A. & Howey, D. A. Gaussian Process Regression for <italic>In Situ</italic> Capacity Estimation of Lithium-Ion Batteries. *IEEE Transactions on Industrial Informatics* **15**, 127-138, doi:10.1109/tii.2018.2794997 (2019).

12 Li, W. *et al.* Online capacity estimation of lithium-ion batteries with deep long short-term memory networks. *J. Power Sources* **482**, doi:10.1016/j.jpowsour.2020.228863 (2021).

13 Roman, D., Saxena, S., Robu, V., Pecht, M. & Flynn, D. Machine learning pipeline for battery state-of-health estimation. *Nature Machine Intelligence* **3**, 447-456, doi:10.1038/s42256-021-00312-3 (2021).

14 Dubarry, M. & Beck, D. Analysis of Synthetic Voltage vs. Capacity Datasets for Big Data Li-ion

Diagnosis and Prognosis. *Energies* **14**, doi:10.3390/en14092371 (2021).

15 Li, X., Yuan, C. & Wang, Z. State of health estimation for Li-ion battery via partial incremental capacity analysis based on support vector regression. *Energy* **203**, doi:10.1016/j.energy.2020.117852 (2020).

16 Lyu, Z., Gao, R. & Li, X. A partial charging curve-based data-fusion-model method for capacity estimation of Li-Ion battery. *J. Power Sources* **483**, doi:10.1016/j.jpowsour.2020.229131 (2021).

17 Pan, W. *et al.* A health indicator extraction and optimization for capacity estimation of Li-ion battery using incremental capacity curves. *Journal of Energy Storage* **42**, doi:10.1016/j.est.2021.103072 (2021).

18 Zhang, C. *et al.* An adaptive battery capacity estimation method suitable for random charging voltage range in electric vehicles. *IEEE Transactions on Industrial Electronics*, 1-1, doi:10.1109/tie.2021.3111585 (2021).

19 Stroe, D.-I. & Schaltz, E. Lithium-Ion Battery State-of-Health Estimation Using the Incremental Capacity Analysis Technique. *IEEE Transactions on Industry Applications* **56**, 678-685, doi:10.1109/tia.2019.2955396 (2020).

20 Guo, P., Cheng, Z. & Yang, L. A data-driven remaining capacity estimation approach for lithium-ion batteries based on charging health feature extraction. *J. Power Sources* **412**, 442-450, doi:10.1016/j.jpowsour.2018.11.072 (2019).

21 Eddahech, A., Briat, O. & Vinassa, J.-M. Determination of lithium-ion battery state-of-health based on constant-voltage charge phase. *J. Power Sources* **258**, 218-227, doi:10.1016/j.jpowsour.2014.02.020 (2014).

22 Chen, C., Wei, Z. & Knoll, A. C. Charging Optimization for Li-ion Battery in Electric Vehicles: A Review. *IEEE Transactions on Transportation Electrification* (2021).

23 Brenna, M., Foiadelli, F., Leone, C. & Longo, M. Electric vehicles charging technology review and optimal size estimation. *Journal of Electrical Engineering & Technology*, 1-14 (2020).

24 Tomaszewska, A. *et al.* Lithium-ion battery fast charging: A review. *ETransportation* **1**, 100011 (2019).

25 Sørensen, D. R. *et al.* Fatigue in High-Energy Commercial Li Batteries while Cycling at Standard Conditions: An In Situ Neutron Powder Diffraction Study. *ACS Applied Energy Materials* **3**, 6611-6622 (2020).

26 Zhu, J. *et al.* Investigation of lithium-ion battery degradation mechanisms by combining differential voltage analysis and alternating current impedance. *J. Power Sources* **448**, 227575 (2020).
